# The potential role of miRNAs and regulation of their expression in the development of mare endometrial fibrosis

**DOI:** 10.1038/s41598-023-42149-3

**Published:** 2023-09-24

**Authors:** Anna Wójtowicz, Tomasz Molcan, Karolina Lukasik, Ewelina Żebrowska, Klaudia Pawlina-Tyszko, Artur Gurgul, Tomasz Szmatoła, Monika Bugno-Poniewierska, Graca Ferreira-Dias, Dariusz J. Skarzynski, Anna Szóstek-Mioduchowska

**Affiliations:** 1 Department of Reproductive Immunology and Pathology, Institute of Animal Reproduction and Food Research of Polish Academy of Sciences, Tuwima 10, 10-748 Olsztyn, Poland; 2 Molecular Biology Laboratory, Institute of Animal Reproduction and Food Research, Polish Academy of Sciences, Tuwima 10, 10-748 Olsztyn, Poland; 3 Department of Animal Molecular Biology, National Research Institute of Animal Production, Cracow, Poland; 4 Center for Experimental and Innovative Medicine, University of Agriculture in Krakow, Cracow, Poland; 5 Department of Animal Reproduction, Anatomy and Genomics, The University of Agriculture in Krakow, Cracow, Poland; 6 Faculty of Veterinary Medicine, CIISA - Center for Interdisciplinary Research in Animal Health, University of Lisbon, Lisbon, Portugal; 7 Associate Laboratory for Animal and Veterinary Sciences (AL4AnimalS), Lisbon, Portugal; 8 Department of Reproduction and Clinic of Farm Animals, Faculty of Veterinary Medicine, Wroclaw University of Environmental and Life Sciences, Wroclaw, Poland

**Keywords:** Gene expression analysis, Genomic analysis, High-throughput screening, Biological techniques, Molecular biology

## Abstract

Mare endometrial fibrosis (endometrosis), is one of the main causes of equine infertility. Despite the high prevalence, both ethology, pathogenesis and the nature of its progression remain poorly understood. Recent studies have shown that microRNAs (miRNAs) are important regulators in multiple cellular processes and functions under physiological and pathological circumstances. In this article, we reported changes in miRNA expression at different stages of endometrosis and the effect of transforming growth factor (TGF)-β1 on the expression of the most dysregulated miRNAs. We identified 1, 26, and 5 differentially expressed miRNAs (DEmiRs), in categories IIA (mild fibrosis), IIB (moderate fibrosis), and III (severe fibrosis) groups compared to category I (no fibrosis) endometria group, respectively (P_adjusted_ < 0.05, log2FC ≥ 1.0/log2FC ≤  − 1.0). This study indicated the potential involvement of miRNAs in the regulation of the process associated to the development and progression of endometrosis. The functional enrichment analysis revealed, that DEmiRs target genes involved in the mitogen-activated protein kinases, Hippo, and phosphoinositide-3-kinase (PI3K)-Akt signalling pathways, focal adhesion, and extracellular matrix-receptor interaction. Moreover, we demonstrated that the most potent profibrotic cytokine—TGF-β1—downregulated novel-eca-miR-42 (P < 0.05) expression in fibroblasts derived from endometria at early-stage endometrosis (category IIA).

## Introduction

Mare endometrial fibrosis (endometrosis) is one of the major reasons for equine infertility^1–3^. Endometrosis leads to alterations in endometrial structure and function, causing changes in the uterine environment and early pregnancy loss^1,4^. It is a degenerative chronic condition of the mare uterus, described mainly as a fibrotic process developing around the endometrial glands and in the stroma. Moreover, endometrosis is associated with inflammatory infiltrates as well as the dilation of endometrial glands and lymphatic vessels in equine endometrium. There are two terms: endometrosis and endometriosis used in the medical literature, which is defined differently and should not be used interchangeably. Endometriosis means extrauterine implantation of endometrial tissue and refers to women^5^, whereas the term endometrosis means changes in the mare’s uterus previously referred to as chronic degenerative endometritis^1^.

Despite the increasing number of studies focusing on endometrosis, its pathogenesis remains not completely understood^6^. Its root cause seems to be repeated and chronic inflammation, associated with the age, numbers of foaling and breeding^1,3,7^. Studies on endometrosis focused mainly on the role of proinflammatory cytokines, neutrophils, and prostaglandins^8–14^. There are multiple signalling pathways associated with the development of fibrotic diseases, including transforming growth factor (TGF)-β1 signalling. TGF-β1 is a main profibrotic cytokine^15^, important in processes associated with the development of mare endometrosis^11^. Previously, TGF-β1 was shown to increase expression of extracellular matrix (ECM) components in mare endometrial fibroblasts cultured in vitro as well as to stimulate fibroblast proliferation^8^. The profibrotic mechanisms of TGF-β1 action are regulated by cross-talk with other signalling pathways, and epigenetics mechanisms, including microRNAs (miRNAs)^15^.

Nowadays, epigenetic determinants start to evoke great interest. Body of evidence indicates a meaningful role of miRNAs at the site of injury and fibrosis and disturbed miRNA expression along with the progression of liver, heart, skin, kidney, and lung fibrosis^16^. However, to the best of our knowledge, there is no information concerning the regulatory role of miRNAs and their regulators in the development of endometrosis in the mare. Recent studies revealed alteration in miRNAs expression in mare endometrial fibroblasts cultured in vitro treated with lipopolysaccharides (LPS)^17^. The stimulation with LPS imitates bacterial infection, thus LPS induced changes in miRNAs expression indicates possible regulatory role of miRNAs in inflammation, which is one of the major causes of endometrosis. Moreover, another study showed, that LPS stimulation changes of TGF-β signaling in human keratinocytes^18^.

miRNAs belong to a group of small (~ 22 nucleotides long) non-coding RNA molecules, that regulate gene expression at the post-transcriptional level, leading to mRNA degradation and translational inhibition. One miRNA can regulate multiple gene transcripts, which is why they are so significant expression regulators^19^. Recent studies identified many novel miRNAs associated with multiple organs fibrosis^20–22^. Moreover, miRNAs expression dysregulated with the progression of fibrosis seem to be susceptible to therapeutic modulation. Thus, miRNAs are in the area of interest for targeted therapies. The miRNA families can be described as either pro- or antifibrotic, depending on their target mRNA^16^.

Through understanding of the core molecular signalling pathways of fibrosis among multiple organs is the essence of further development of novel diagnostics methods and therapeutics. The main aim of the present study was to describe the miRNAs expression signature and thus elucidate the potential molecular processes regulated by differentially expressed miRNAs (DEmiRs) at every stage of endometrosis of mares being in the follicular phase of the estrous cycle. The follicular phase of the estrous cycle was selected based on our previous study, which showed the most changes in the transcriptome associated with fibrosis-related processes^14^. Further, we investigated, if the most potent profibrotic cytokine—TGF-β1 affects the expression of the most dysregulated in endometrial fibroblasts.

This study aims at describing mare endometrial miRNA profile at each stage of endometrosis and finding potential regulation of their expression by the most potent profibrotic cytokine—TGF-β1.

## Results

We conducted next-generation sequencing (NGS) to characterize the miRNAs expression profile in the endometrium of mares in the follicular phase of the estrous cycle at every stage of endometrosis. The miRNA profiling results were submitted to the NCBI BioProject under the accession number PRJNA880660. From 26,304,055 to 2,650,974 reads in individual samples were obtained. Subsequent filtering resulted in on average 5,748,485 sequences, which were further mapped to the *Equus caballus* (genome EquCab3.0, Ensembl release 106) mature miRNA sequence from miRBase database 22.1. Most expressed miRNAs were 22 nt long (Supplementary Data 1). In this study, we identified 423 known and 90 novel miRNAs (Supplementary Data 2). Principal component analysis (PCA) performed on the basis of miRNA expression results revealed distinctly separated category IIB samples from category I-derived samples (Supplementary Data 3). The distribution of transcripts, including DEmiRs, identified in the examined samples is depicted in Supplementary Data 4.

### Mare endometrial miRNA expression profile changes along with the severity of endometrosis

The full list of identified DEmiRs between categories IIA, IIB, and III vs*.* I endometria including their mature sequence, log2FC, and P-adjusted is provided in Supplementary Data 5.

**Figure 1:**
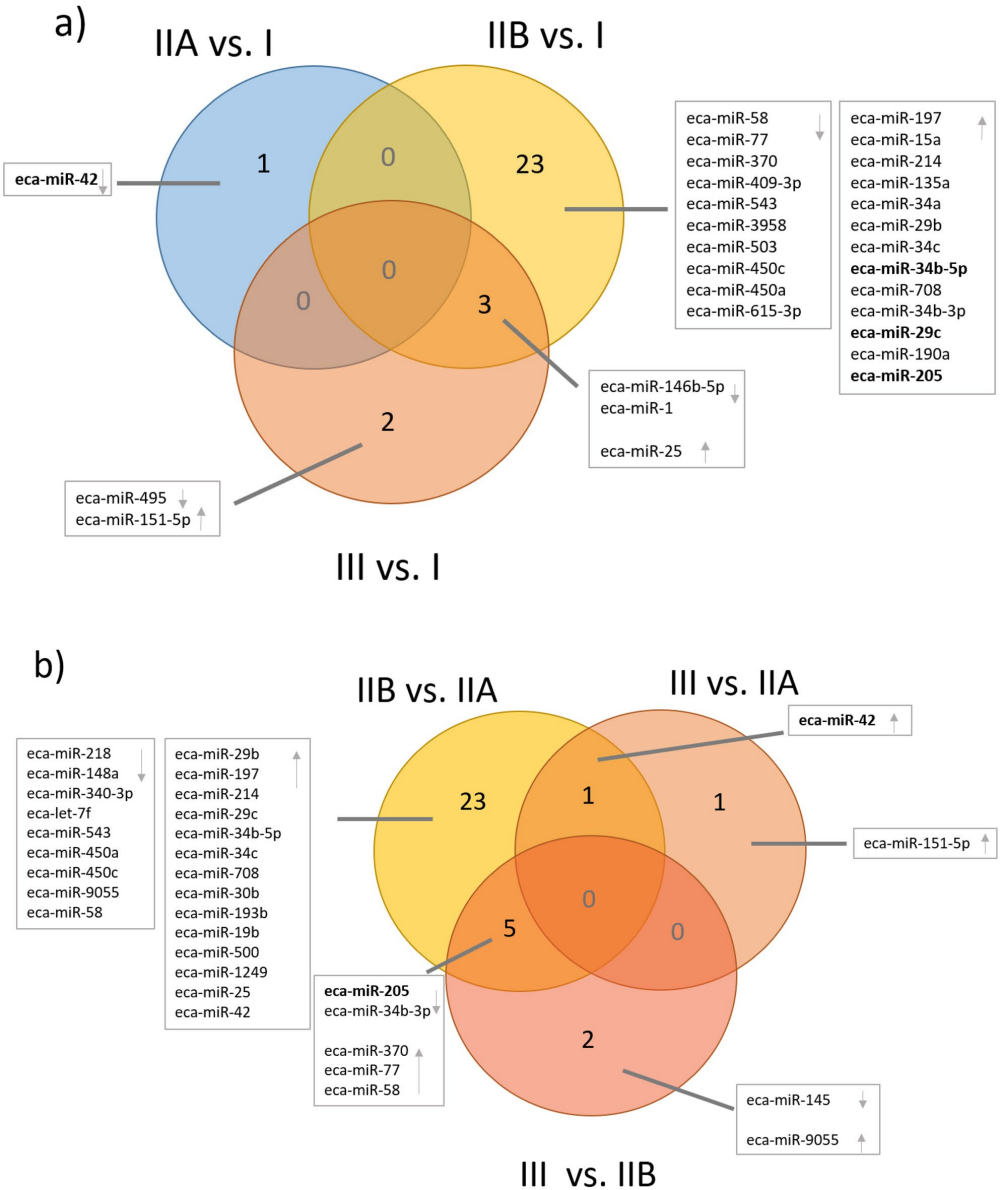
Differentially expressed miRNA (DEmiRs) identified between different categories of mare endometrium with endometrosis. (**a**) Category IIA (mild fibrosis), IIB (moderate fibrosis), and III (severe fibrosis) vs. I (no fibrosis) pairs and (**b**) IIA, IIB, and III pairs presented as Venn diagram. Arrows indicate downregulation or upregulation of identified DEmiRs in particular comparisons.

In category IIA vs. category I endometria, we identified one DEmiR—novel-eca-miR-42 (Fig. 1a). We found most DEmiRs in category IIB endometria compared to category I endometria. Briefly, we identified 26 DEmiRs (Fig. 1a) of which 12 were downregulated (including eca-miR-146b-5p, novel-eca-miR-58, novel-eca-miR-77, eca-miR-370, and eca-miR-409-3p), while 14 were upregulated (including eca-miR-34a, eca-miR-29b, eca-miR-34c, eca-miR-34b-5p, eca-miR-34b-3p, eca-miR-29c, eca-miR-190a, eca-miR-205, and novel eca-miR-25). In category III vs. I endometria, five DEmiRs were identified, three of which were downregulated (eca-miR-146b-5p, eca-miR-495 and eca-miR-1), and two were upregulated (eca-miR-151-5p and eca-miR-25; Fig. 1a).

In categories IIB vs. IIA, we identified 29 DEmiRs; 11 of them were downregulated and 19 were upregulated (Fig. 1b). In categories III vs. IIA endometria, two DEmiRs were identified; one downregulated (eca-miR-151-5p) and one upregulated (novel-eca-miR-42). In comparison between categories III and IIB endometria, we identified seven DEmiRs: three down- (eca-miR-145, eca-miR-205, eca-miR34b-3p) and four upregulated (eca-miR-370, eca-miR-77, eca-miR-58, eca-miR-9055).

**Figure 2:**
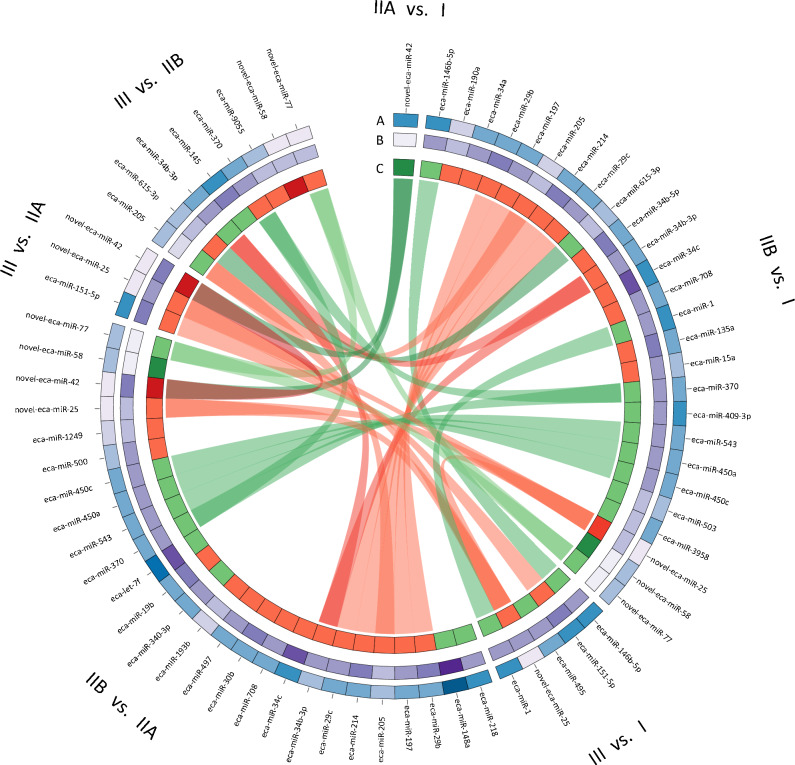
Circos plot illustrating the expression profile of differentially expressed miRNA (41 DEmiRs; P-adjusted ≤ 0.05 and log2FC ≥ 1.0 or log2FC ≤  − 1.0) in mare endometrium category I (no fibrosis), IIA (mild fibrosis), IIB (moderate fibrosis), and III (severe fibrosis). Track A (blue) represents log-transformed mean of Reads Per Kilobase Million (RPKM) values of control samples (maximum value = 7.384); Track B (violet) represents log-transformed mean of RPKM values of treatment samples (maximum value = 7.217); Track C (green–red) depicts fold changes in the miRNA expression level between two respective comparison (IIA compared to I, IIB compared to I, III compared to I, IIB compared to IIA, III compared to IIA or III compared to IIB), where the brightest green depicts the − 27.175-fold change and the brightest red the 27.103-fold change; Links in the middle of the circular plot connect these miRNA, expression of with significantly differed between any two sets of compared condition; Gene names originated from miRBase (v. 22.1).

The circos plot illustrates the expression profile of DEmiRs in endometrial samples in both comparisons between fibrous endometria (categories IIA, IIB, and III) vs. non-fibrous (category I) as well as between tissue with different stages of endometrosis (Fig. 2).

**Figure 3:**
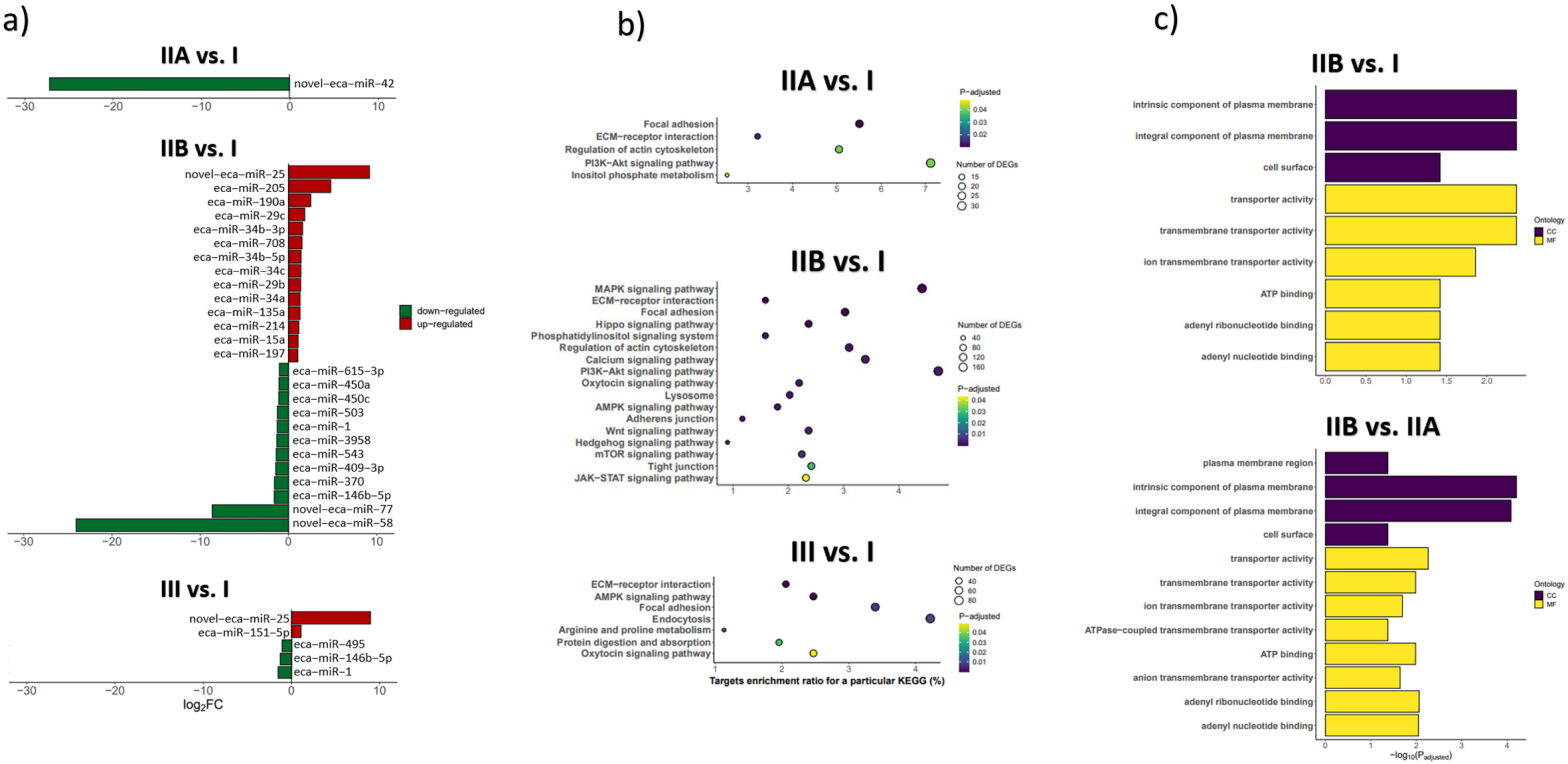
Differentially expressed miRNAs (DEMiRs) in mare endometrium with I (no fibrosis), IIA (mild fibrosis), IIB (moderate fibrosis), and III (severe fibrosis) category, KEGG, and GO pathway analysis concerning DEmiRs target genes. (**a**) Differentially expressed miRNAs and the corresponding fold changes. (**b**) KEGG pathways of miRNA target genes. The y axis is pathway categories. The x axis refers to pathway enrichment. (**c**) Gene Ontology (GO) analysis of miRNA target genes. The y axis is GO categories. The x axis refers to GO enrichment. *CC* cellular components, *MF* molecular functions.

Among all DEmiRs, the most downregulated were: novel-eca-miR-42, eca-miR-77, eca-miR-58, and eca-miR-146b-5p, while among most upregulated were: eca-miR-25, eca-miR-205, eca-miR-190a, eca-miR-29c, and eca-miR-34b-3p (Fig. 3a, Supplementary Data 5). Therefore, for the next steps of the study four miRNAs with the most changed expression and available TaqMan probes (novel-eca-miR-42, eca-miR-29c, eca-miR-34b-3p, and eca-miR-205) were selected.

### DEmiRs enriched molecular and cellular functions important during the development of fibrosis

The analysis of target genes of the identified DEmiRs enabled the prediction of signalling pathways through KEGG pathway analysis^23–25^. The number of target genes identified for particular DEmiRs in selected comparisons among different endometrosis categories is presented in Supplementary Data 6, while the full list of DEmiRs target genes is included in Supplementary Data 7. We identified seven (IIA vs. I), 12 (IIB vs. IIA), 58 (IIB vs. I), 65 (IIB vs. IIA), two (III vs. IIA), and nine (III vs. IIB) KEGG pathways enriched by DEmiRs identified in the indicated comparisons.

The enriched KEGG pathways shared by categories IIA vs. I and IIB vs. I endometria, include focal adhesion, extracellular matrix (ECM)-receptor interaction, MAPK and PI3-Akt signalling pathways, and regulation of actin cytoskeleton (Fig. 3b). The unique KEGG pathways enriched by IIB vs. I DEmiRs are, among others: Hippo, calcium, oxytocin, AMPK, Wnt, Hedgehog, mTOR, and JAK-STAT signalling pathways. The full list of KEGG enriched pathways with indicated DEmiRs and their target genes is provided in Supplementary Data 8. Selected KEGG pathways in category IIA vs. I and IIB vs. I as well as III vs. IIB endometria enriched by the identified DEmiRs in depicted comparisons are presented in Fig. 3b.

Gene ontology (GO) analysis was used to predict the biological processes, cellular components (CC) and molecular functions (MF) that were the gene product enrichments of identified DEmiRs (Fig. 3c). In categories IIB vs. I and IIB vs. IIA endometria, we indicated nine and 12 GO categories, respectively. Our results showed the following most significantly enriched GO terms in category IIB vs. I endometria among CC: intrinsic and integral component of plasma membrane and cell surface, while cell surface, transmembrane, transporter, and ion transmembrane transporter activity, ATP, adenyl ribonucleotide and nucleotide binding among MF category (Supplementary Data 9). In category IIB vs. IIA endometria, the analysis revealed plasma membrane region (CC) as well as anion transmembrane and ATPase-coupled transporter activity (MF; Supplementary Data 9).

### Validation of selected DEmiRs by qRT-PCR

To validate the miRNA-Seq results, four miRNAs (novel-eca-miR-42, eca-miR-29c, eca-miR-34b-3p, and eca-miR-205) were selected for qRT-PCR. A comparison of DEmiRs log2fold change and P-value obtained after qPCR and P-adjusted after NGS analyses is presented in Table 1. Analysis confirmed decreased novel-eca-miR-42 expression in endometria category IIA compared to category I (P < 0.05) and increased eca-miR-205 expression in category IIB tissue compared to category I endometria (P < 0.05) (Table 1).

**Table 1:** Results of RT-qPCR validation of NGS results. Category IIA (mild fibrosis) vs. category I (lack of fibrosis) endometria; category IIB (moderate fibrosis) vs. category I endometrium.

miRNA	RT-qPCR		NGS	
	Log2FC	P-value	Log2FC	P-adjusted
Category IIA vs. I				
*eca-miR-42*	− 7.44	0.0124	− 27.18	5.27E−09
Category IIB vs. I				
*eca-miR-29c*	0.85	0.2564	1.84	2.84E−02
*eca-miR-34b-3p*	1.29	0.3259	1.43	3.50E−02
*eca-miR-205*	7.34	0.0142	4.79	1.20E−04

**Figure 4:**
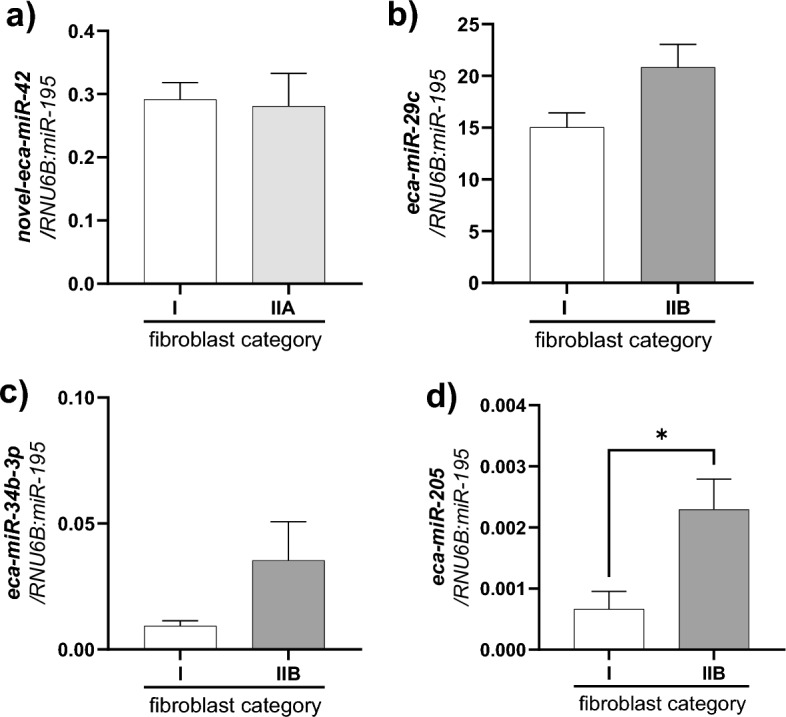
The comparison of the expression of selected for validation DEmiRs (**a**—*novel-eca-miR-42*, **b**—*eca-miR-29c*, **c**—*eca-miR-34b-3p*, **d**—*eca-miR-205*) in mare endometrial fibroblasts (n = 4) cultured in vitro isolated from endometria category I (no fibrosis), IIA (mild fibrosis), and IIB (moderate fibrosis). Results are presented as arbitrary units of relative expression means. Asterisks denote statistical differences (*P < 0.05, nonparametric Mann–Whitney U-test).

We also compared the expression level of selected DEmiRs in cultured endometrial fibroblasts derived from categories IIA and IIB compared to category I endometria samples. In cultured fibroblasts derived from category IIB endometria, eca-miR-205 expression was upregulated compared to fibroblasts derived from category I endometria (P < 0.05; Fig. 4d). In the case of the expression levels of novel-eca-miR-42 (P > 0.05; Fig. 4a), eca-miR-29c (Fig. 4b), and eca-miR-34b-3p (P > 0.05; Fig. 4c) there were no statistically significant differences in mare endometrial fibroblasts isolated from endometria category IIA (P > 0.05; Fig. 4a) and IIB (P > 0.05; Fig. 4b,c) cultured in vitro*.*

### TGF-β1 decreases novel-eca-miR-42 expression in mare endometrial fibroblasts

**Figure 5:**
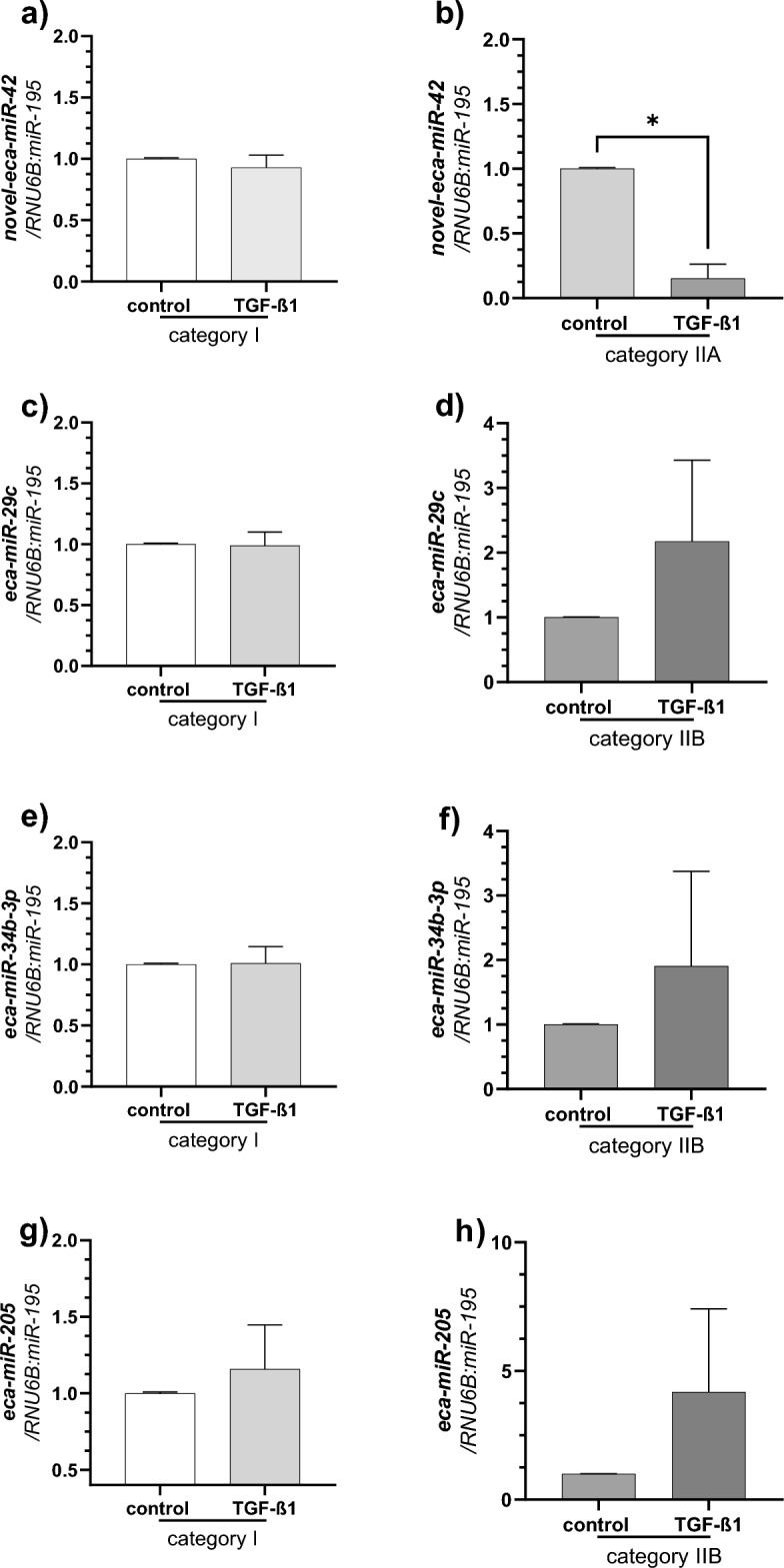
The effect of TGF-β1 treatment (10 ng/ml) on *eca-miR-42* (**a,b**), *eca-miR-29c* (**c,d**), *eca-miR-34b-3p* (**e,f**), *eca-miR-205* (**g,h**) expression in in vitro cultured fibroblast (n = 4 per group) derived from endometria with category I (no fibrosis; **a,c,e,g**), IIA (mild fibrosis; **b**), and IIB (moderate fibrosis; **d**,**f**,**h**). Results are presented as a fold change. Asterisks denotes statistical difference (*P < 0.05, nonparametric Mann–Whitney U-test).

The in vitro experiment with the use of the most potent profibrotic cytokine—TGF-β1, revealed that after 24 h of treatment, the expression level of novel-eca-miR-42 was decreased in fibroblasts derived from category IIA endometria compared to non-treated fibroblasts (P < 0.05; Fig. 5b). Interestingly, TGF-β1 did not affect the expression of novel-eca-miR-42 in mare endometrial fibroblasts isolated from endometria without fibrosis (category I, P > 0.05; Fig. 5a). It indicates a possible influence of TGF-β1 on novel-eca-miR-42 expression levels at the early stage of endometrosis development. There was no statistically significant influence of TGF-β1 on the expression of eca-miR-29c (P > 0.05; Fig. 5c,d), eca-miR-34b-3p (P > 0.05; Fig. 5e,f), and eca-miR-205 (P > 0.05; Fig. 5g,h) in cultured fibroblasts derived from category I and IIB endometria (P > 0.05); however, we observed the tendency of TGF-β1 to increase their expression in fibroblasts isolated from endometria category IIB.

## Discussion

A growing body of evidence demonstrates disturbances in miRNA expression across multiple tissues fibrosis. However, to date, the miRNA expression profile—miRNAome of mare endometrium with endometrosis has not been described. Thus, we compared the endometrial miRNA expression profiles at every stage of endometrosis development. Further analyses showed most DEmiRs in category IIB compared to I endometria, indicating the possible importance of miRNAs in the development of mare endometrial fibrosis with the greatest importance in the advanced stage. Different DEmiRs were revealed along with the advancement of fibrosis, which may potentially regulate the expression of genes involved in such processes as focal adhesion, ECM-receptor interaction, and PI3-Akt signalling in mare endometrium.

The expression regulation of important in fibrotic disorders genes can be an essential mode of action of the identified DEmiRs in the development of endometrosis. Identified in the current study DEmiRs (inter alia: eca-miR-29b, eca-miR-29c, eca-miR-205, eca-miR-34c, novel-eca-miR-42, eca-miR-503) are associated with fibrosis via their target genes. These genes belong to ECM components: collagens (including COL1A1, COL5A1, COL6A3, COL8A2, COL9A1, and COL24A1), fibronectin (FN), elastin (ELN), and laminin (LAMA-1, -2, -3, -4, and -5). Moreover, also matrix metalloproteinases (MMP2, -13, -17, -19) and their tissue inhibitor (TIMP4) are targeted by the identified DEmiRs. Interestingly, these DEmiRs possibly regulate also immune response by affecting expression levels interferons (IFNA1, IFNA2, IFNK), interleukins (IL1A, IL10, IL17A, IL17B, IL18, IL27), interleukins receptors (IL1R1, IL6R, IL10RA, IL13RA2, IL17RA), and interleukin 4-induced gene-1 (IL4I1).

In mild fibrosis (category IIA), we found one, highly downregulated DEmiR—novel-eca-miR-42 compared to non-fibrous endometrium (category I). The target genes of the miRNA are engaged in pathways significant for fibrotic diseases, including focal adhesion, ECM-receptor interaction, PI3K-Akt signalling pathway, and regulation of actin cytoskeleton^26–31^. Moreover, we showed that TGF-β1 treatment decreased novel-eca-miR-42 expression in endometrial fibroblasts derived from category IIA endometria, in contrast to fibroblasts from category I endometria. miR-1388 (*Danio rerio, Ornithorhynchus anatinus*, and *Taeniopygia guttata*), which shares sequence with novel-eca-miR-42 with, was shown to inhibit NF-κB signalling pathway^32^, which in turn plays a role in liver fibrosis^33^. A recent study, concerning gene expression of NF-κB-dependent pathway in mare endometrium, indicated most changes in the follicular phase of the oestrous cycle^34^, confirming the possible important role of NF-κB-pathway in mare endometrosis progression. To the best of our knowledge, no research indicated the significance of miR-42 and its regulation by TGF-β1 in fibrotic disorders so far. Our findings suggest the contribution of novel-eca-miR-42 to signalling pathways associated with the development of fibrosis, and the regulation of its expression by TGF-β1. Thus, the downregulation of its expression in category IIA endometria as well as after TGF-β1 treatment of fibroblasts may denote miR-42 importance in molecular mechanisms at the early stage of fibrosis development. Since the foaling rate even decreases to 50% in IIA category of endometrosis mares^35^ it is important to find key drivers of initial and further development fibrosis in mare endometrium.

The highest number of DEmiRs (14 up- and 12 downregulated) were found in the endometrial tissue in category IIB compared to category I. It suggests that miRNAs are important regulators of processes taking part in the moderate stage of fibrosis. In the current study, eca-miR-58 was the most downregulated in category IIB vs. I endometria. To the best of our knowledge, there is no information on the action of this molecule in processes related to the development of fibrosis. However, the KEGG enrichment analysis revealed the potential role of novel-eca-miR-58 in the regulation of key processes associated with the development of fibrosis, such as Hippo, Wnt, MAPK, JAK-STAT, Hedgehog, and mTOR signalling pathways, as well as ECM-receptor interaction and focal adhesion. A recent study indicated the cross-species anti-inflammatory and pro-autophagia role of *Salvia miltiorrhiza*-derived sal-miR-58^36^. As the level of novel-eca-miR-58 was significantly reduced in our study, it can be hypothesized, that it may lead to the upregulation of proinflammatory and profibrotic genes expression. Restoring its level may be a potential treatment in fibrotic diseases^16^.

Among the identified in the present study DEmiRs, miR-29 (eca-miR-29b, eca-miR-29c) and miR-34 (eca-miR-34a, eca-miR-34b-3p, eca-miR-34b-5p, eca-miR-34c) families were previously described as drivers of various organ fibrosis^37^. The importance of the role of miR-29 and miR-34 in the development of fibrosis in many organs may be indicated by the attempts to target their action for the treatment of fibrotic diseases^38,39^. The miR-29 family was identified in this study, and seems to have multiple regulatory functions, controlling cell differentiation^40^, proliferation, migration^41^, and apoptosis^42^. Moreover, miR-29 family has even been called the master regulator of fibrosis due to their high impact on the development of fibrosis^16^. Our findings indicate the potential role of the members of miR-29 family in the development of fibrosis via regulation of multiple signalling pathways, including MAPK^43–45^, Hippo^46,47^, phosphatidylinositol, calcium, PI3K-Akt^28–31,48^, AMPK^49^, and Wnt^47^, as well as focal adhesion^50^, and ECM-receptor interaction. All these signalling pathways and processes were described as crucial in the progression of fibrosis. Previous studies indicated also a control role of miR-34 family over cell proliferation, migration^51^, and apoptosis^52^. The significant role of miR-34 family members in renal^53^, cardiac^54^, and hepatic fibrosis^55^ was also demonstrated.

In categories IIB and III (severe fibrosis), compared to category I endometria, we identified, out of others, the following DEmiRs: eca-miR-151-5p, eca-miR-370, novel-eca-miR-25, and eca-miR-205. Interestingly, our findings indicate the potential involvement of mentioned DEmiRs in the regulation of genes involved in oxytocin (OXT) signalling, such as *OXT receptor*, *prostaglandin-endoperoxide synthase 2*, *phospholipase C beta 1* and *nitric oxide synthase 3.* Proper OXT responsiveness and signalling are crucial in the proper regulation of the oestrous cycle, and thus embryo implantation, and pregnancy in mares^56^. Additionally, previous research showed that miR-151-5p, miR-370, miR-25, miR-205^57–60^ are connected with processes accompanying fibrosis. Since, endometrosis is related to the decline of foaling rate^1^, altered both OXT signalling and pro-fibrotic action of DEmiRs may be important causes of reduced fertility in mares with endometrosis. However, these findings need further studies.

In category III vs. I endometria, we identified two downregulated miRNAs: eca-miR-146b-5p and eca-miR-49. These DEmiRs have already been described as related to fibrosis spread in both in vivo as well as in in vitro studies^61–63^. miR-146b-5p was shown to regulate IL-17-dependent activation of NF‐κB^64^, while miR-49 was found to regulate important in fibrotic disorders: focal adhesion kinase signalling^65^. Interestingly, a recent study found the possible role of miR-146b-5p in attenuating dermal fibrosis^62^. The authors of this study showed that miR-146b-5p may repress Platelet-derived growth factor receptor α-induced profibrotic activities, thus decreasing the development of skin fibrosis. Another study implicated the important role of miR-146b-5p in cardiac remodelling^66^.

The mechanisms involved in the development of fibrosis are tissue-specific; however, molecular processes between tissues share many similarities^67^. Finding molecules and biological pathways that are commonly differed in various organs undergoing fibrotic changes are essential to understand molecular background of these changes and design effective therapeutic strategies against fibrosis^68^. Mare endometrial fibrosis occurs naturally, in contrast to experimental animal models in which fibrosis is chemically induced^69^. What is more, endometrial biopsies can be taken routinely by veterinarians multiple times from each mare, what does not cause significant discomfort to the animals. Moreover, it is possible to culture in vitro cells and tissues taken from mares with various advancement of fibrosis to study molecular mechanisms. Thus, the use of the horse as a model of organ fibrosis would reduce the sacrifice of animals at the initial stages of research. Altogether, mare endometrosis could serve as a model to study the mechanism of the development of fibrotic diseases in humans.

Increasing number of studies concerning miRNAs as crucial players in the development of fibrosis indicate the need to study their mechanism of action in fibrotic diseases. However, the action of miRNAs is not unambiguous due to the characteristic feature of miRNAs^16^, as each miRNA can regulate the expression of many genes due to the imperfect nature of miRNA binding to its target mRNA. In turn, the expression of each gene can be regulated by multiple miRNAs. This nature of miRNAs therefore offers a plethora of opportunities for each miRNA to regulate the expression of target genes. This characteristic miRNAs regulatory action creates both many opportunities and causes difficulties both in analysing the action of miRNAs and in targeting their action^70^. These limitations, however, cannot overcome the plethora of possibilities of using miRNAs both in the search for markers of fibrosis and in the treatment that would target miRNAs action. To the date, despite limitations, miRNAs as biomarkers of various diseases are growing field of research^71,72^. Nevertheless, the studies of miRNAs as biomarkers of disease severity are still on its early stage due to numerous limitations. The results of these studies must be critically interpreted, and perhaps in the near future, miRNA-based markers and therapies will form a significant part of diagnosis and treatment^73^.

In conclusion, the current study showed changes in the miRNA expression signature at different stages of endometrosis, suggesting the potential role of miRNAs in the development and progression of mare endometrial fibrosis. In category IIA vs. I endometria, we identified one downregulated miRNA (novel-eca-miR-42), in category IIB vs. I—26 DEmiRs (14 up- and 12 downregulated), while in category III vs I—five DEmiRs (2 up- and 3 downregulated). The functional enrichment analysis revealed that identified DEmiRs may regulate multiple processes, such as focal adhesion, ECM-receptor interaction, Hippo and PI3K-Akt signalling pathways, which can contribute to endometrosis development and progression. Moreover, we demonstrated that the most potent profibrotic cytokine—TGF-β1 decreased the expression of novel-eca-miR-42 in fibroblasts derived from category IIA endometria. Likewise, we showed that along with the development of endometrosis, the miRNA expression becomes disturbed and some of DEmiRs can regulate the expression of genes associated with OXT signalling. These results may suggest impaired response to OXT in endometria with endometrosis, which can be regulated by miRNAs. Further in-depth understanding of the roles of miRNAs in processes associated with endometrosis initiation and progression may help guide the diagnosis and create targeted therapies in the future.

## Materials and methods

### Ethics approval

All experimental protocols were approved by the Local Ethics Committee for Experiments on Animals in Olsztyn, Poland (Approval No. 60/2014/DTN). All methods were carried out in accordance with relevant guidelines and regulations. All methods are reported in accordance with ARRIVE guidelines for the reporting of animal experiments.

### Experimental design

#### Experiment 1: endometrial miRNA expression profiling at different stages of endometrial fibrosis in the mare

To perform miRNA expression profiling in mare endometrium at each stage of endometrosis severity, miRNA Next Generation Sequencing (NGS) was performed. For this purpose, total RNA was isolated from mare endometria at follicular phase of oestrous cycle (n = 9 per category I, IIA, IIB, and III, n = 36 in total). Then, the NGS analysis followed by bioinformatic analyses were performed.

#### Experiment 2: the effect of TGF-β1 on selected DEmiRs expression

To determine if expression of selected DEmiRs is regulated by most potent profibrotic cytokine—TGF-β1, in vitro experiment was performed. Thawed fibroblasts (n = 4 per category I, IIA and IIB, n = 12 in total) were seeded on T75 cm^2^ flasks and cultured in 38.0 °C in 5% CO_2_. After reaching 90% of confluence, fibroblasts were passaged and seeded on 6-well plates. After reaching 80% confluence, the culture medium was replaced with fresh Dulbecco’s Modified Eagle’s Medium/Nutrient Mixture F-12 Ham (DMEM/Ham’s F-12; D2906; Sigma-Aldrich, St. Louis, MO, USA) supplemented with 0.01% of AA solution (A5955; Sigma-Aldrich, St. Louis, MO, USA), ascorbic acid (100 ng/ml; A4544; Sigma-Aldrich, St. Louis, MO, USA) and 0.1% (w/v) BSA (A1470, Sigma-Aldrich, St. Louis, MO, USA), and the cells were incubated at 38.0 °C in 5% CO_2_. After serum starvation, cells were treated with the vehicle (culture medium alone) or TGF-β1 (10 ng/ml; human recombinant; 100-21; PeproTech, Rocky Hill, NJ, USA) for 24 h. The dose of TGF-β1 was chosen based on previous experiments^8^. After treatment, the cells were washed with sterile PBS (P4417; Sigma-Aldrich, St. Louis, MO, USA), trypsinized, flash-frozen in liquid nitrogen, and stored in − 80 °C for subsequent RNA extraction and RT-qPCR analysis. The RNA isolation and RT-qPCR were performed as described below. The analysis of expression of eca-miR-42 was done in fibroblasts, cultured with and without TGF-β1, derived from categories I and IIA, while expression of eca-miR-29c, eca-miR-34b-3p, and eca-miR-205 was done in categories I and IIB. Moreover, we compared the expression level of selected DEmiRs in mare endometrial fibroblasts cultured in vitro between categories IIA (novel-eca-miR-42) or IIB (eca-miR-29c, eca-miR-34b-3p, and eca-miR-205) and category I.

### Materials

#### Tissue collection for NGS analysis, fibroblast isolation and histological analysis

Procedures were reviewed and accepted by the Local Ethics Committee for Experiments on Animals in Olsztyn, Poland (Approval No. 60/2014/DTN). The study was conducted between April and June 2016 (Exp.1) and 2021 (Exp. 2). Sixty-four clinically healthy, normally cycling cold-blooded mares (weighing 500 ± 100 kg) at age 2–20 were used in this study. The animals were slaughtered to obtain meat as part of routine breeding as slaughter animals. Endometrial tissue samples were collected from mare endometrium in each category of endometrosis. The endometria were collected 5 min after the slaughter. The follicular phases of the estrous cycle were identified based on P_4_ analysis of blood plasma and the macroscopic observation of ovaries. The follicular phase was characterized by the absence of an active CL and the presence of follicles of various sizes, but always > 35  mm in diameter, with a concentration of P_4_ < 1  ng/ml as described previously^74^. Endometrial tissue was excised, rinsed with cold sterile RNAse-free saline solution, divided, and put in RNAlater (R0901; Sigma-Aldrich, St. Louis, MO, USA) and stored in − 80 °C for further RNA analyses and in 4% formaldehyde for histological examination. After haematoxylin–eosin staining, endometria were retrospectively assigned to Kenney and Doig classification^35^. This classification takes into account primarily the presence of inflammation and fibrosis, but also endometrial atrophy and dilatation of lymphatic vessels. Endometrium without pathological changes was classified as category I (Supplementary Data 10). Category IIA includes endometrium with mild to moderate inflammation, with few dilated lymphatic vessels. A small degree of fibrosis affects individual branches of the mammary glands, and there are no fibrotic gland sockets in 4 adjacent visual fields. Category IIB includes moderately inflamed endometrium with numerous dilated lymphatic vessels. Fibrosis is more severe than category IIA, with 2–4 fibrotic glandular pockets in 4 adjacent visual fields. Changes affect up to 60% of the uterine glands. Category III includes an endometrium with massive inflammation and greatly dilated lymphatic vessels. The fibrosis is massive, 5 or more fibrotic glands are found in 4 adjacent visual fields. Changes affect over 60% of the uterine glands in the most severe endometrosis stage^75^.

#### The isolation and culture of fibroblasts from mare endometrium

The fibroblasts were isolated from mare endometrium (n = 4 per category I, IIA and IIB, n = 12 in total), cultured, and passaged, as previously described with modifications^8^. The cells were counted using a haemocytometer. The viability of endometrial cells was higher than 95% as assessed by the trypan blue exclusion test.

The dispersed cells were seeded separately at a density of 5 × 10^5^ viable cells/ml and cultured at 38.0°C in a humidified atmosphere of 5% CO_2_ in the air. To purify the fibroblast population, the medium was changed 18 h after plating, by which time selective attachment of fibroblasts had occurred. The fibroblast homogeneity was confirmed using immunofluorescent staining for vimentin based on the protocol described recently^76^. The purity of fibroblast after isolation was around 96%. After reaching 90% of confluency, the cells were cryopreserved as described previously^77^. After haematoxylin–eosin staining endometria were retrospectively assigned as a category I, IIA, IIB, or III according to the Kenney and Doig classification^35^.

### Experimental procedures

#### RNA extraction

##### RNAextraction for NGS analysis

Total RNA was extracted using the Direct-zol RNA MiniPrep kit (Zymo Research, Irvine, California, USA) according to the manufacturer’s protocol. RNA concentration and quality were measured using a NanoDrop 2000 spectrophotometer (Thermo Fisher Scientific, Waltham, Massachusetts, USA) and a 2200 TapeStation instrument (Agilent, Santa Clara, USA); only samples with ratio A260 nm/230 nm between 1.8 and 2.2 and RNA integrity number (RIN) values greater than 8.0 were used. The pooling of samples was performed to reduce the experimental costs and the variability among individual samples and at the same time have more individuals within an experiment.

##### RNA extraction and RT-qPCR analysis for NGS results validation and in vitro experiment

To validate the NGS results, four DEmiR were selected for qRT-PCR. The most changes novel-eca-miR-42 (Assay ID: 008434), and miRNAs whose roles in different tissues fibrosis were described previously: eca-miR-29c^78,79^ (Assay ID: 000587), eca-miR-34b-3p^51^ (Assay ID: 002618), and miR-205^59^ (Assay ID: 000509) were selected. Due to the prolonged period between the NGS analysis and RT-qPCR analysis, different RNA templates were used for NGS and RT-qPCR analyses. Additionally, RT-qPCR was used to determine the effect of TGF-β1 on the expression of the selected DEmiRs in mare endometrial fibroblasts in vitro.

Total RNA was extracted either from endometrial tissue (Experiment 1, n = 4 per category I, IIA, IIB, and III according to Kenney and Doig^35^, n = 16 in total) or cultured fibroblasts (Experiment 2, n = 12) using mirVana isolation kit (AM1560, Invitrogen, Carlsbad, CA, USA).

The concentration and quality of total RNA were determined spectrophotometrically. The ratio of absorbance at 260 and 280 nm (A260/280) was approximately 2. Total RNA (10 ng) was reverse transcribed using a TaqMan MicroRNA Reverse Transcription Kit (4,366,597; Invitrogen) and specific RT primers according to the manufacturer’s directions. The cDNA was stored at − 20 °C until qPCR. Further, qPCR was performed in a final volume of 20 µl, using 1.33 µl (1.9 ng) of cDNA, 1 µl of specific primers with probes, 10 µl TaqMan Universal PCR Master Mix II (4,440,049; Applied Biosystems; Thermo Fisher Scientific, Wilmington, NC, USA), and 7.67 µl nuclease-free water, on 96-well plates. All samples were run in duplicates. Amplification was performed with initial denaturation for 10 min at 95 °C, followed by 48 cycles of 15 s at 95 °C and 60 s at 60 °C with ViiA 7 Real-Time PCR System (Agilent Technologies, Waltham, MA, USA). As a negative control, nuclease-free water instead of template cDNA was used. The efficiency of miR-42, miR-29c, miR-34b-3p, and miR-205 were 80%, 80%, 83%, and 83%, respectively.

The qPCR data were analyzed by the method described previously^80^. It is a method for quantifying qPCR results using calculations based on the kinetics of individual PCR reactions without the need for the standard curve, independent of any assumptions or subjective judgments, which allows direct calculation of efficiency and CT. The relative concentration of mRNA (R0) for each target and reference miRNAs (selected based on our previous study: RNU6B and miR-195^17^) was calculated using the equation R0 = 1/(1 + E)Ct, where, E is the average gene efficiency and Ct is the cycle number at the threshold. The relative gene expression was calculated as R0target gene/R0reference gene and was expressed in arbitrary units. NormFinder^81^ was used to confirm the selection of the most stable reference genes for the normalization of the results.

#### RT-qPCR data analysis

All data were tested using Gaussian distribution using the Shapiro–Wilk test (GraphPad Software version 9; GraphPad, San Diego, CA). Whenever the assumptions of normal distribution were not met, nonparametric statistical analyses were done. In experiment 1 the significant differences were determined by the parametric T-test, while in experiment 2, using Mann–Whitney U test. The results were considered significantly different when P < 0.05. GraphPad Prism 9.0 was used both to perform statistical analysis and for generating bar graphs.

#### Small RNA library preparation and NGS sequencing

MicroRNA libraries were prepared with the use of the NEB Next Multiplex Small RNA library Prep Set for Illumina (New England Biolabs, Ipswich, MA, USA) according to the manufacturer’s instructions. Specifically, following 3′ adapter ligation, hybridization of the Reverse Transcription Primer and 5′ adapter ligation, Reverse Transcription and PCR amplification of obtained products were performed. The PCR was prepared with the use of 12 different indexed primers containing a 6 nt long unique sequence, which allows for barcoding the single library and multiplexing of the samples during sequencing. The next step included size selection (Novex 6% TBE PAGE gel; Invitrogen, Carlsbad, CA, USA) of libraries. Subsequently, the quantity of obtained libraries were measured with a Qubit 2.0 Fluorometer (Thermo Fisher Scientific, Waltham, MA, USA), while a 2200 TapeStation instrument (Agilent Technologies, Santa Clara, CA, USA) was used to assess their size. Afterward, the obtained libraries were sequenced on a HiScanSQ sequencing instrument (Illumina, San Diego, CA, USA) according to the manufacturer’s protocol.

#### NGS data analysis

##### Identification of miRNAs

The quality of raw reads was evaluated by means of FASTQC (https://www.bioinformatics.babraham.ac.uk/projects/fastqc/). Low quality reads and adapter sequences were removed by cutadapt software (2.8)^82^ excluding reads: without a 3’-adapter, containing non-determined nucleotides (nt) “N”, shorter than 18 nt or larger than 30 nt. Next, clean reads were mapped to the horse non-coding RNA (ncRNAs; EquCab3.0, Ensembl release 106) with the use of bowtie2 software (2.4.2)^83^. Read mapped to the horse ncRNA were removed from further analysis. The remaining clean reads were then mapped to the mature miRNAs sequences from mirBbase database (22.1)^84^. The miRDeep2 software (0.1.3)^85^ was used to predict new miRNAs from unidentified miRNA reads. After described analysis, due to low number of reads and identified miRNAs, comparing to the remaining samples (median of 3,368,155 reads), one sample from category III (with pooled samples from mares 28, 29 and 30; no of reads: 18 800) was excluded from further analysis. The differentially expressed miRNAs (DEmiRs) and corresponding P-adjusted values were determined by means of R statistical software (4.1.3) using DESeq2 package (1.34.0)^86^. The threshold for the significantly different expression was set at P-adjusted ≤ 0.05 and log2 fold change (log2FC) ≥ 1.0 or log2FC ≤  − 1.0^87,88^. The visual presentation of the results was performed by R software using the ggplot2 package (version 3.3.6).

##### Prediction for targets of miRNAs

The prediction of target genes of the DEmiRs was performed by means of Tools4mirs server^89^ using miRanda^90^, PITA^91^, rna22^92^ and RNAhybrid^93^ tools. The 5′ UTR, CDS and 3′ UTR sequences of horse protein coding genes were used as potential targets. Only miRNA-mRNA pairs predicted in at least three out of four tools were used. Additionally, miRNA-mRNA pairs with the interaction binding free energy above − 8.0 kcal mol^−1^, predicted by PITA and miRanda, were excluded from further analysis.

##### Functional enrichment analysis

Functional analysis of the identified targets genes for differentially expressed miRNAs was performed based on the Gene Ontology (GO) database using clusterProfiler^94^, DOSE^95^, biomaRt^96^ and AnnotationHub^97^ packages of R software, with the established criterion P-adjusted ≤ 0.05. Additionally, the Kyoto Encyclopedia of Genes and Genomes (KEGG) database was used to ascribe identified miRNA targets to particular biological mechanisms and cellular pathways (the established criteria: P-adjusted ≤ 0.05). The KEGG enrichment analysis was performed by clusterProfiler, DOSE and AnnotationHub packages of R software. The visual presentation of the results was performed by R software using ggplot2.

### Supplementary Information

Supplementary Legends.

Supplementary Information 1.

Supplementary Information 2.

Supplementary Information 3.

Supplementary Information 4.

Supplementary Information 5.

Supplementary Information 6.

Supplementary Information 7.

Supplementary Information 8.

Supplementary Information 9.

Supplementary Information 10.

## Data Availability

The datasets used and/or analyzed during the current study are available from the corresponding author upon reasonable request. The miRNA profiling results were submitted to the NCBI BioProject under the Accession Number PRJNA880660.
