# Supplementary material for: The potential role of miRNAs and regulation of their expression in the development of mare endometrial fibrosis
Source: Sci Rep. 2023 Sep 24;13:15938. doi: 10.1038/s41598-023-42149-3 (PMC10518347; doi:10.1038/s41598-023-42149-3)
Supplement: Supplementary file 1 — Supplementary Legends. [file 41598_2023_42149_MOESM1_ESM.docx]

# Supplementary data description:

**Supplementary data 1**. The length distribution and frequency of small RNAs indicated in mare endometrial samples with different stages of endometrosis identified in six *Equus caballus* libraries.

**Supplementary data 2.** Summary of datasets used in the study.

**Supplementary data 3**. The clustering of all samples of the miRNAs identified in the tissue of endometrium at every stage of endometrosis using principal component analysis (PCA). Graphical representation of the first (PC1) and second (PC2) principal components (PC) affecting miRNAs expression pattern in mare endometrial tissue for each comparison of samples of fibrotic endometrium (categories IIA (A), IIB (B), and III (C)) versus non-fibrous endometrium (category I).

**Supplementary data 4**. The volcano plot presents differentially expressed miRNAs (DEmiRs; P-adjusted ≤ 0.05 and log2FC ≥ 1.0 or log2FC ≤ -1.0) in mare endometrium category: I – no fibrosis, IIA – mild fibrosis, IIB – moderate fibrosis, and III – severe fibrosis. DEmiRs are represented by multi-coloured circles, where red colour is upregulated DEmiRs and green downregulated DEmiRs in following comparisons: **a**) IIA vs. I, **b**) III vs. IIA, **c**) IIB vs. I, **d**) III vs. IIB, **e**) III vs. I, **f**) IIB vs. IIA. Grey circles represent all miRNAs identified in the cells with no significant changes.

**Supplementary data 5.** NGS-seq results presenting identified differentially expressed miRNAs (DEmiRs) between categories IIA (mild fibrosis), IIB (moderate fibrosis), and III (severe fibrosis) vs. I (no fibrosis), includes DemiRs names and their sequences.

**Supplementary data 6**. The number of targets genes identified for a particular differentially expressed miRNA (DEmiRs) in following comparisons: **a**) IIA vs. I, **b**) III vs. IIA, **c**) IIB vs. I, **d**) III vs. IIB, **e**) III vs. I, **f**) IIB vs. IIA. Categories of endometrium: I – no fibrosis, IIA – mild fibrosis, IIB – moderate fibrosis, and III – severe fibrosis.

**Supplementary data 7.** List of target genes predicted to be regulated by differentially expressed miRNAs identified in IIA vs. I, IIB vs. I, III vs. I, IIB vs. IIA, III vs. IIA, and III vs. IIB. Categories of mere endometrium: I – no fibrosis, IIA – mild fibrosis, IIB – moderate fibrosis, and III – severe fibrosis.

**Supplementary data 8.** List of KEGG pathways identified differentially expressed miRNAs (DEmiRs) in endometria categories IIA vs. I, IIB vs. I, III vs. I, III vs. IIA, and III vs. IIB of endometrosis. Categories of mare endometrium: I – no fibrosis, IIA – mild fibrosis, IIB – moderate fibrosis, and III – severe fibrosis.

**Supplementary data 9.** Gene ontology (GO) functional enrichment analysis of the target genes predicted to be regulated by differentially expressed miRNAs identified between categories IIB (moderate fibrosis of mare endometrium) vs. I (no fibrosis) and IIB vs. IIA (mild fibrosis).

**Supplementary data 10.** Histological pictures of mare endometrium. Categories of mare endometrium: I – no fibrosis, IIA – mild fibrosis, IIB – moderate fibrosis, and III – severe fibrosis. A) – 5X magnification; B) – 10X magnification.
