# Supplementary figures and images for: The potential role of miRNAs and regulation of their expression in the development of mare endometrial fibrosis
Source: Sci Rep. 2023 Sep 24;13:15938. doi: 10.1038/s41598-023-42149-3 (PMC10518347; doi:10.1038/s41598-023-42149-3)

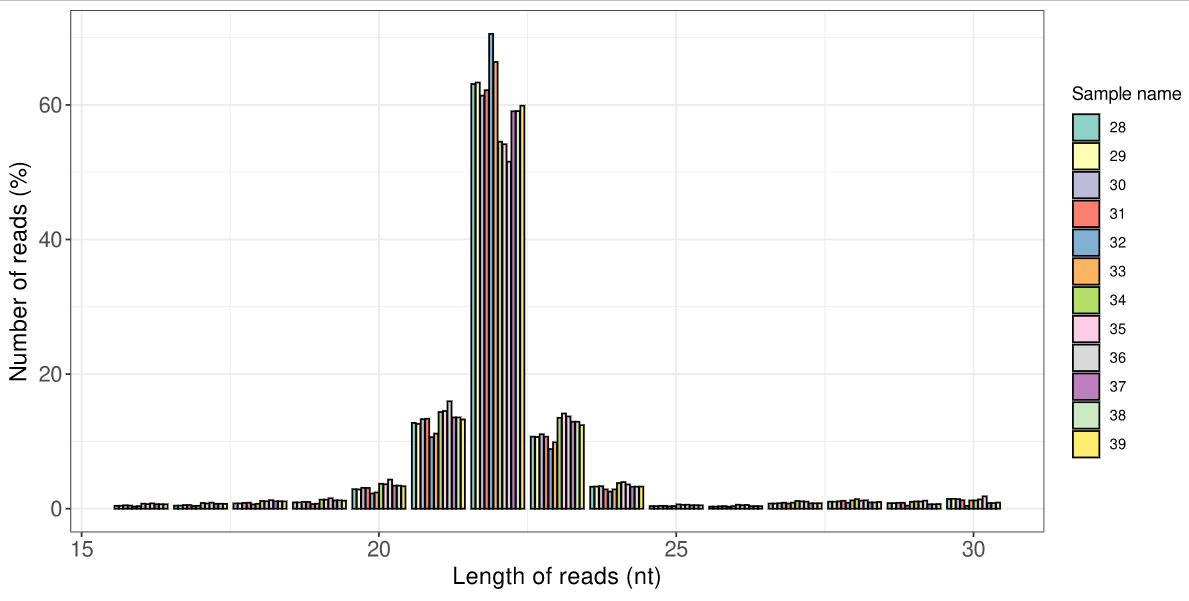

Supplement: Supplementary file 2 — Supplementary Information 1. [file 41598_2023_42149_MOESM2_ESM.png]

A

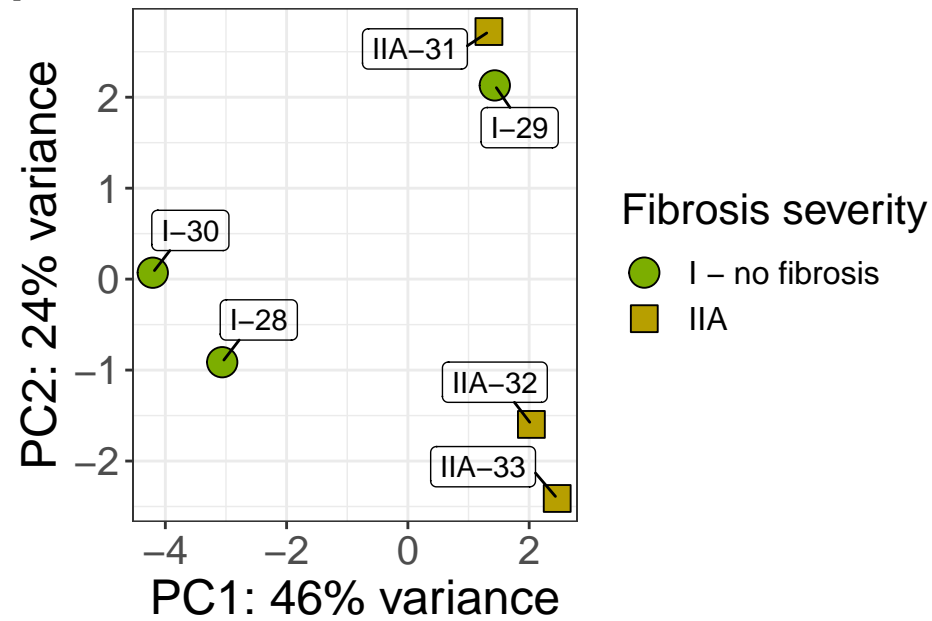

B

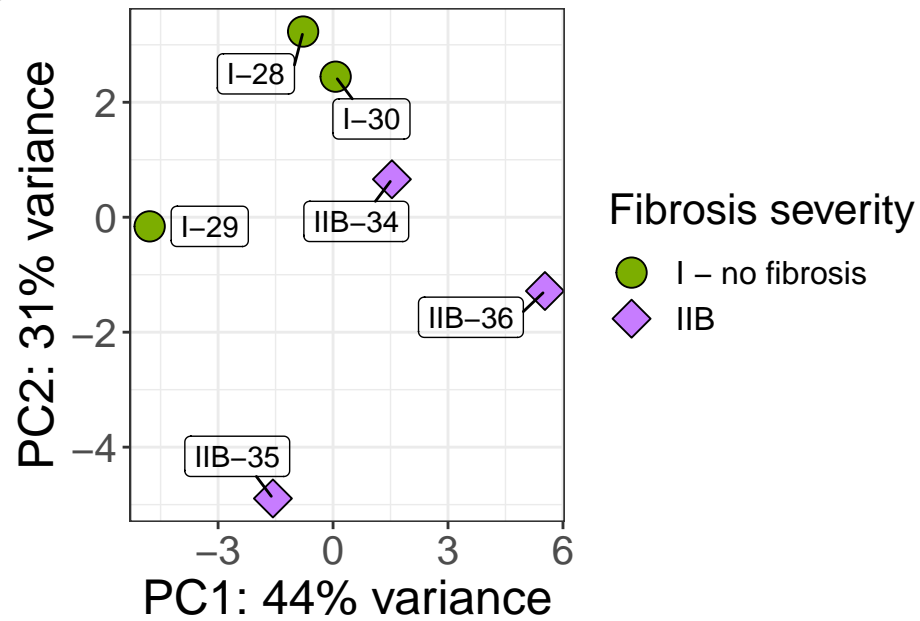

C

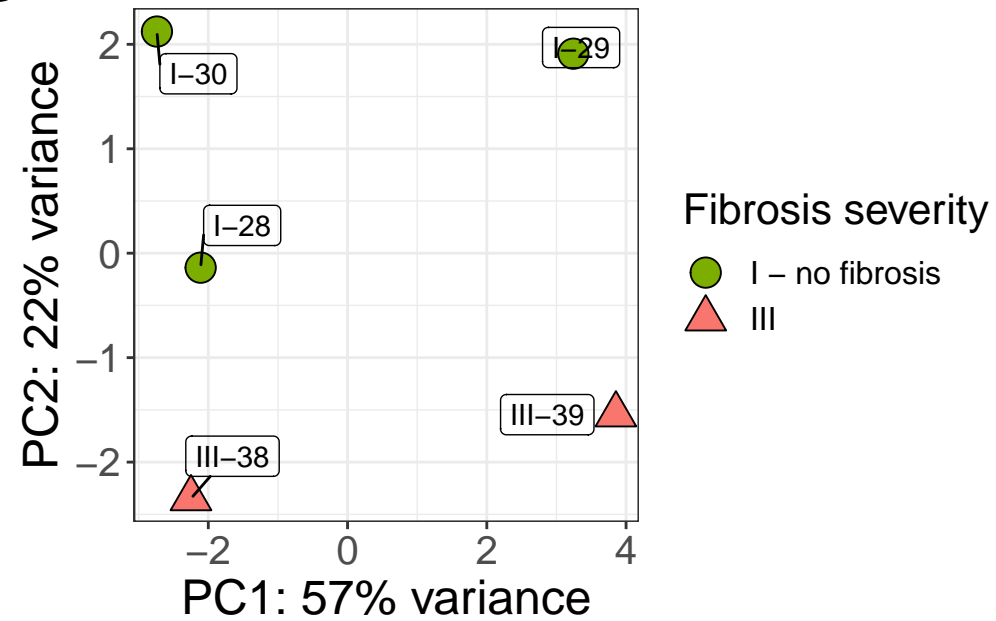

Supplement: Supplementary file 4 — Supplementary Information 3. [file 41598_2023_42149_MOESM4_ESM.pdf]

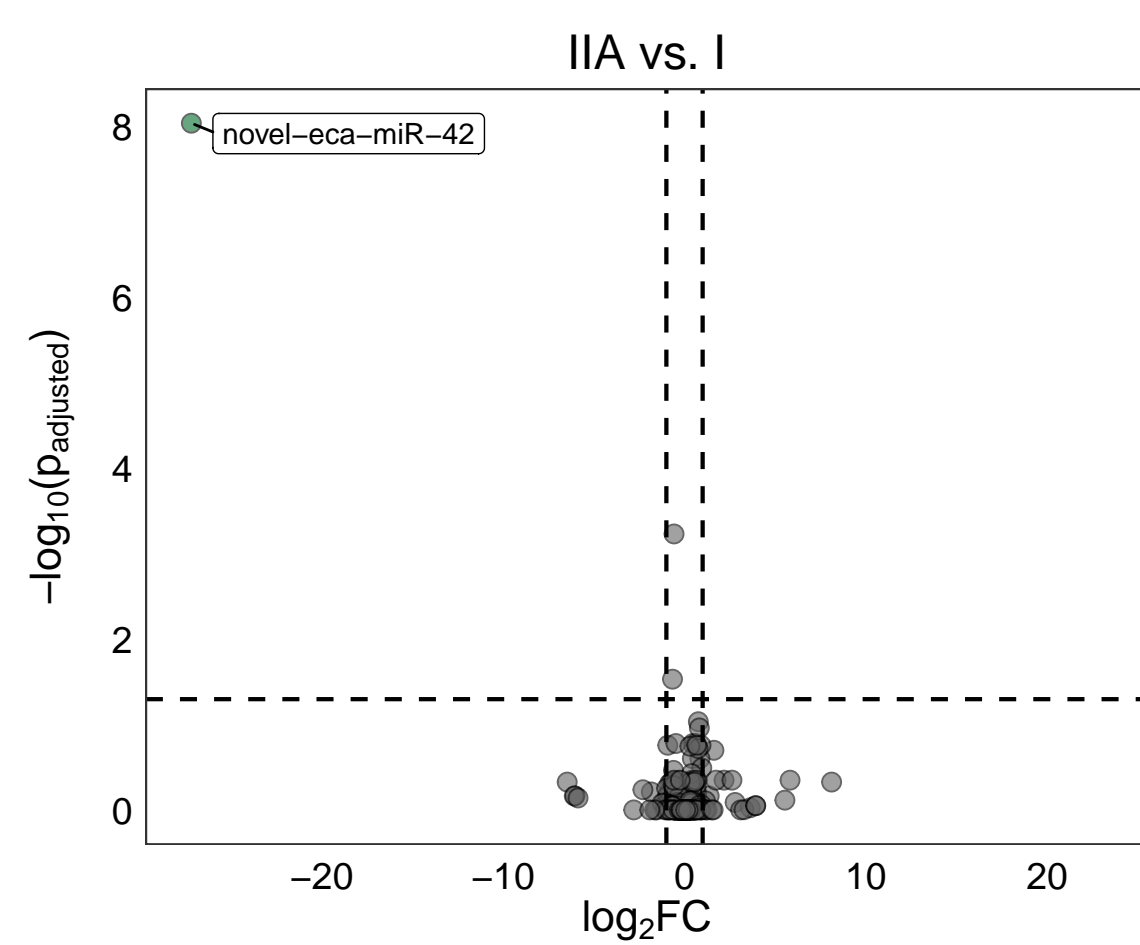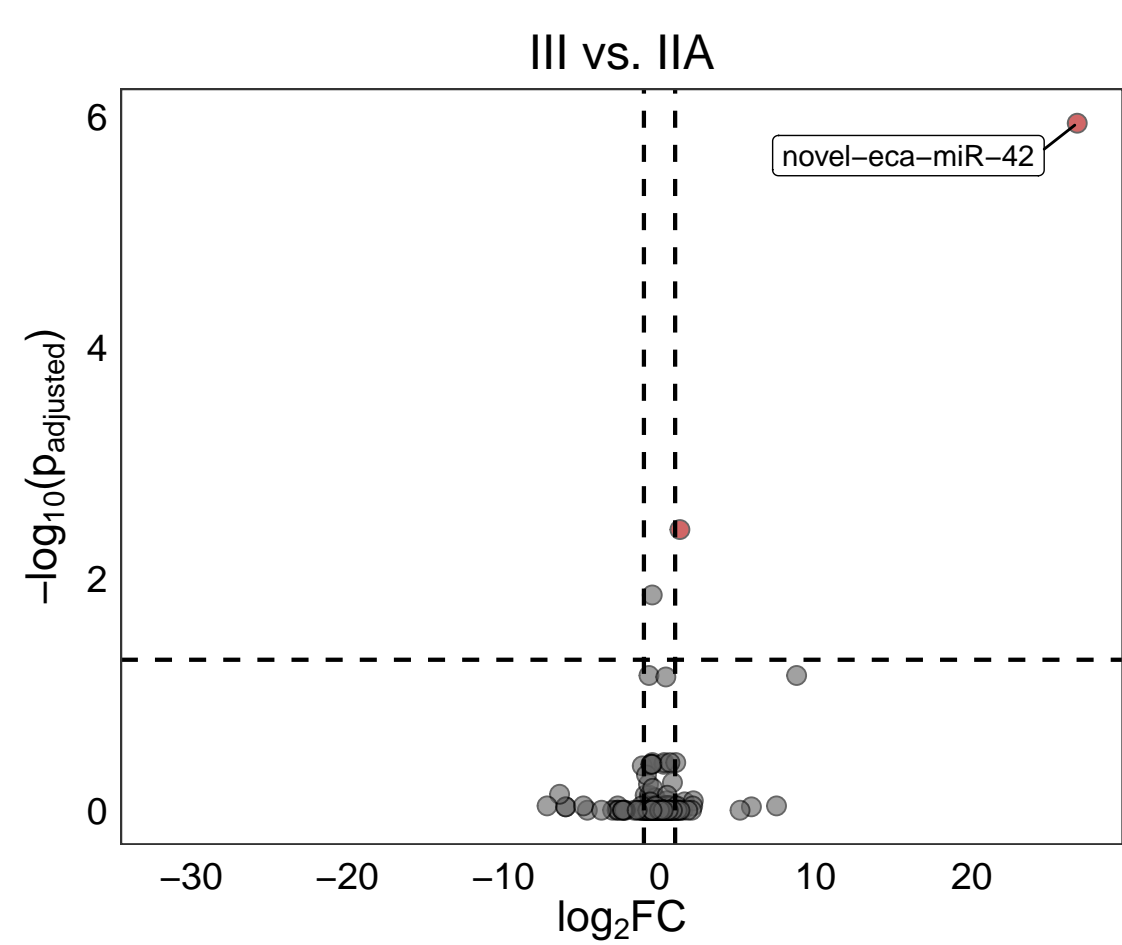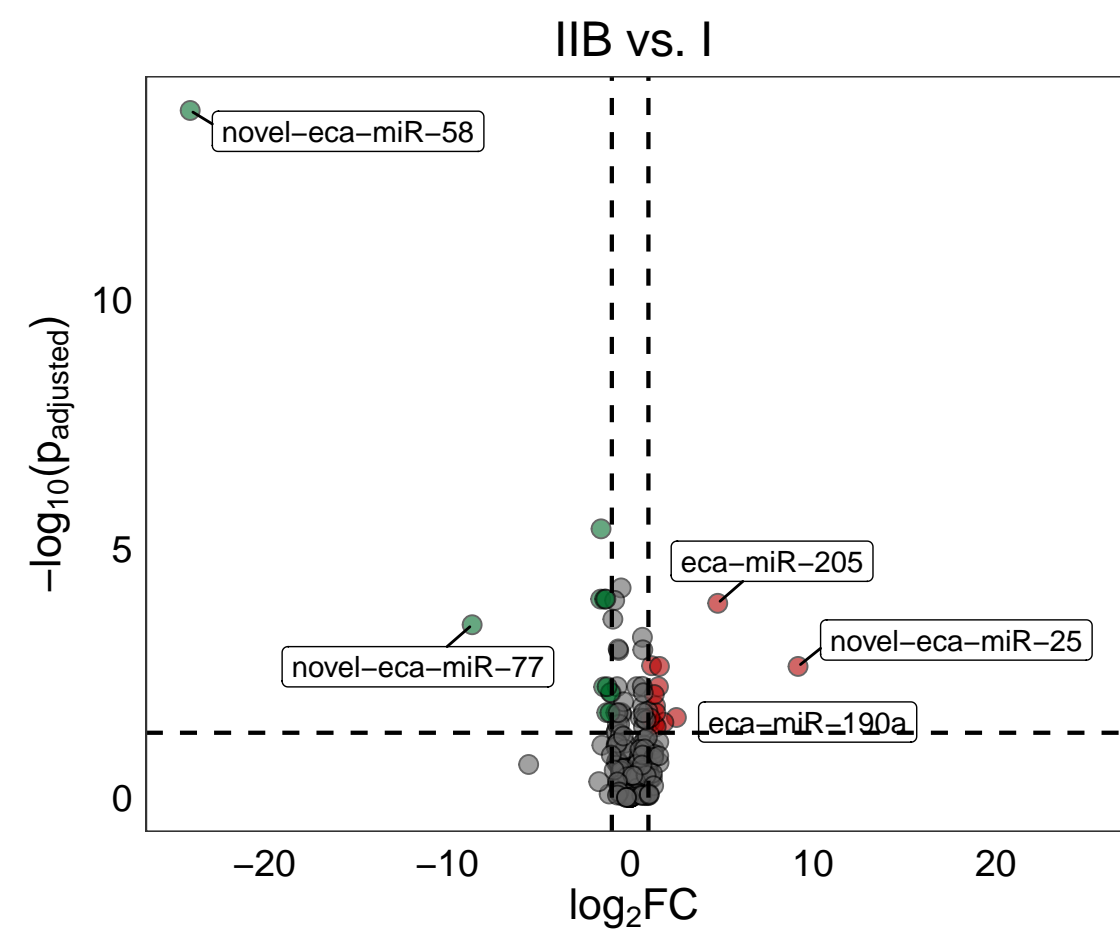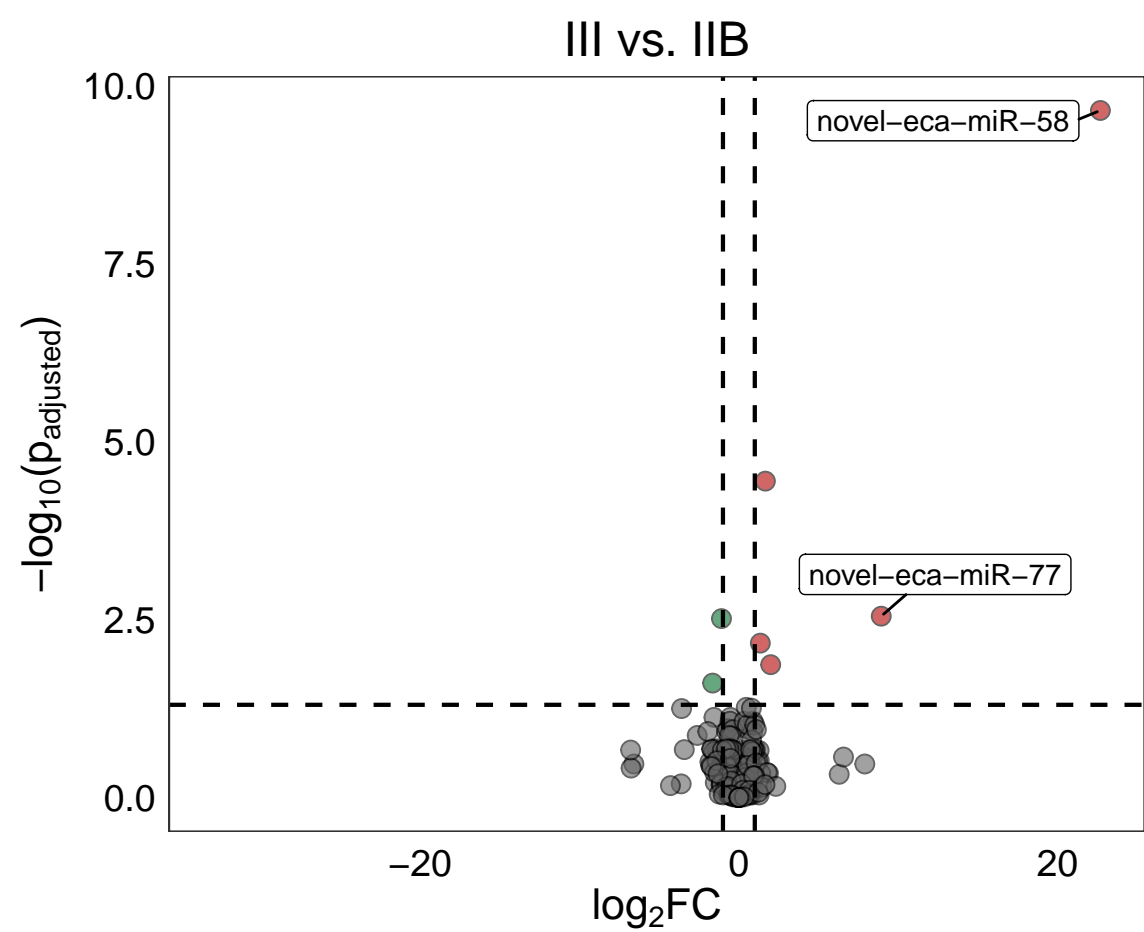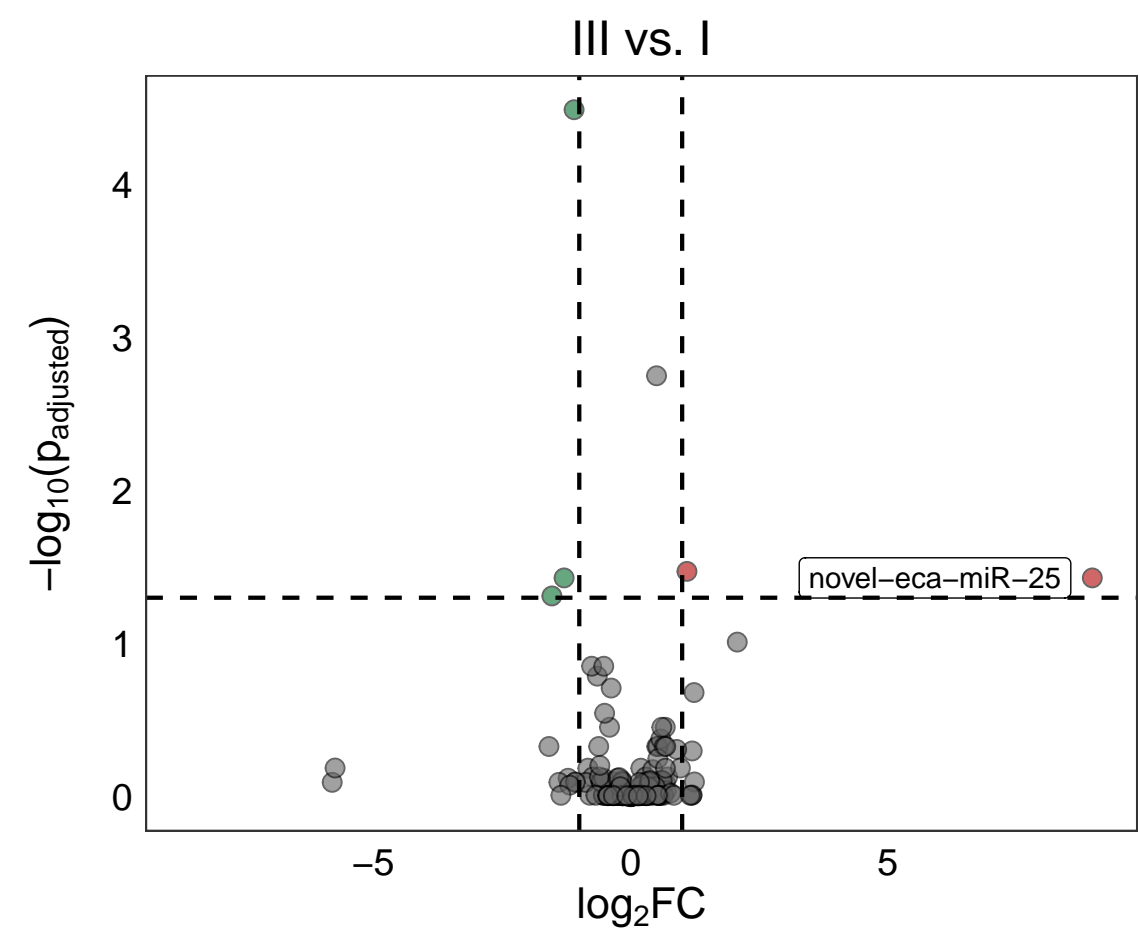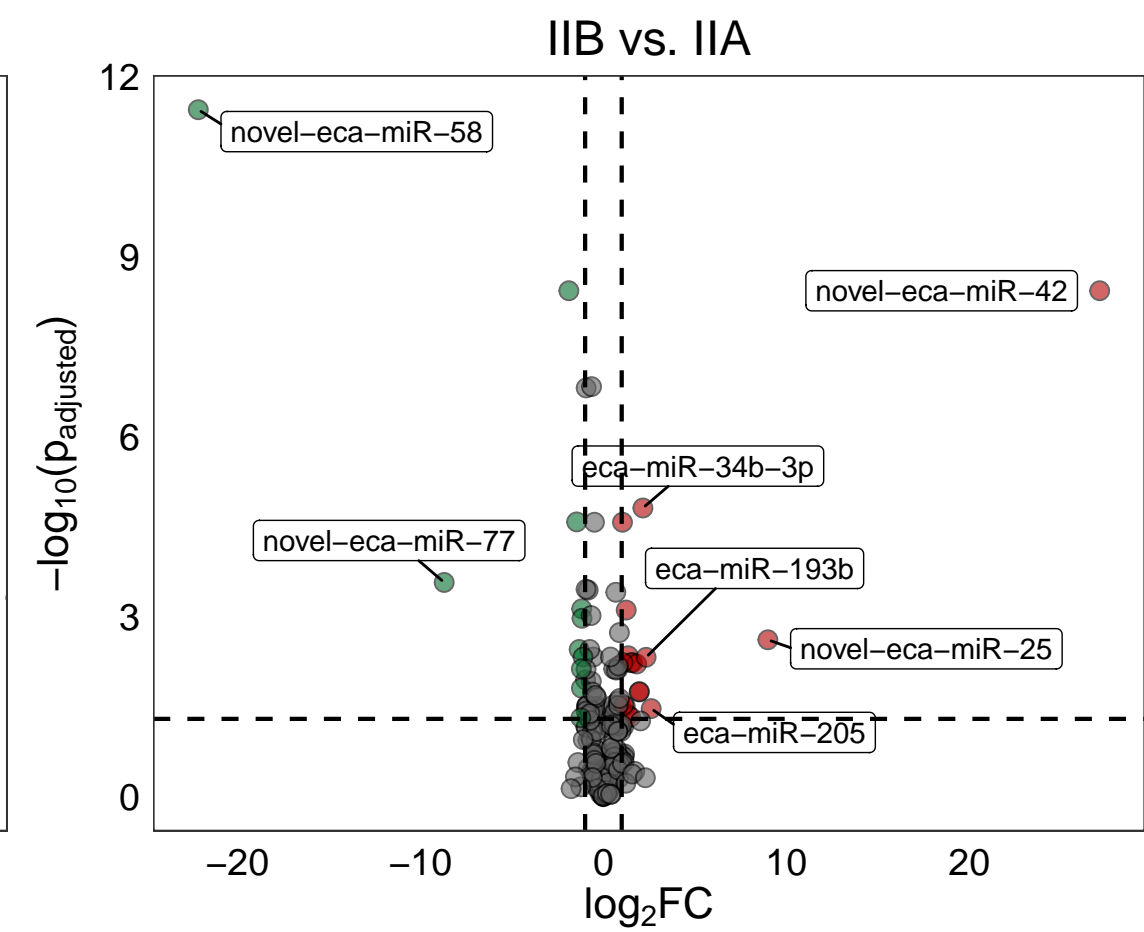

Supplement: Supplementary file 5 — Supplementary Information 4. [file 41598_2023_42149_MOESM5_ESM.pdf]

IIA vs. I

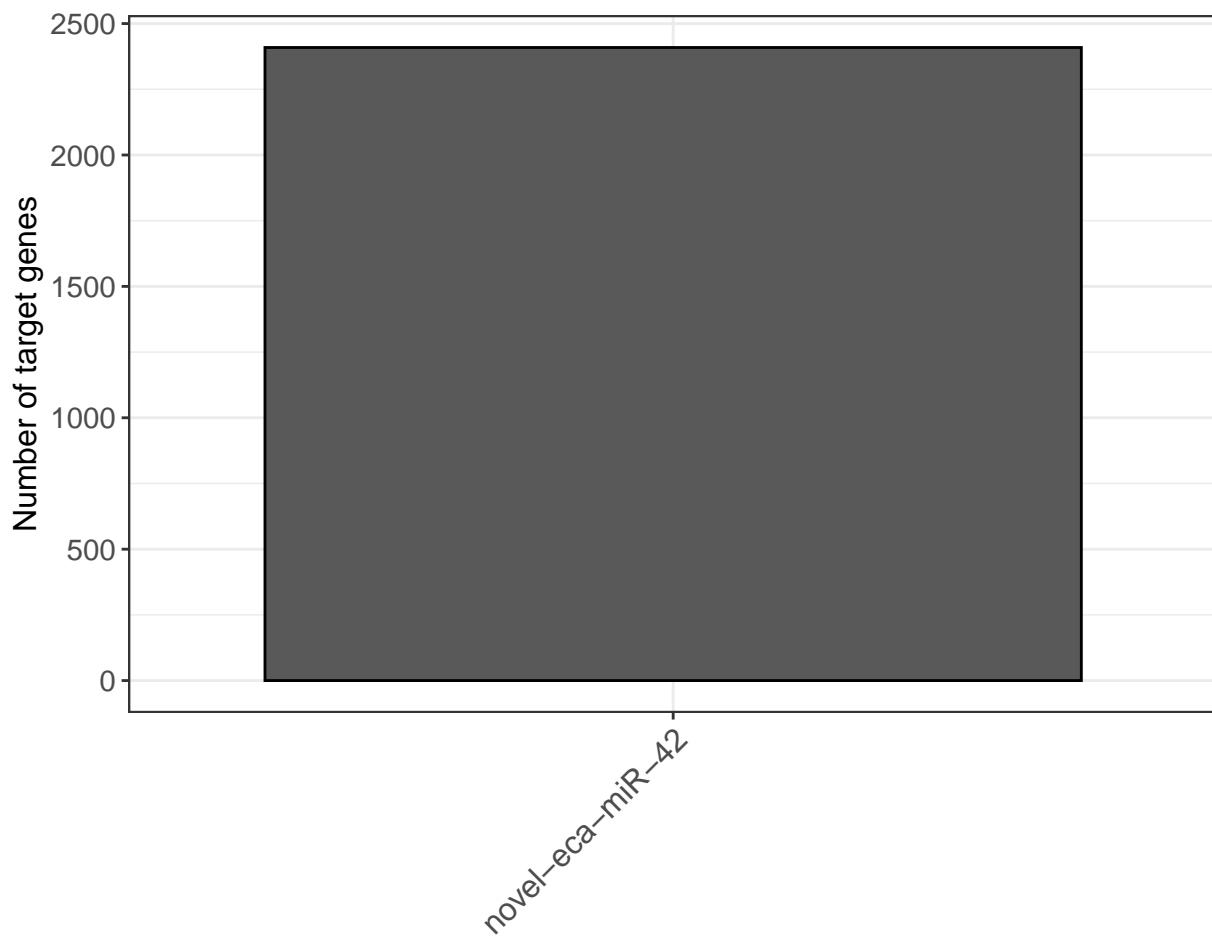

III vs. IIA

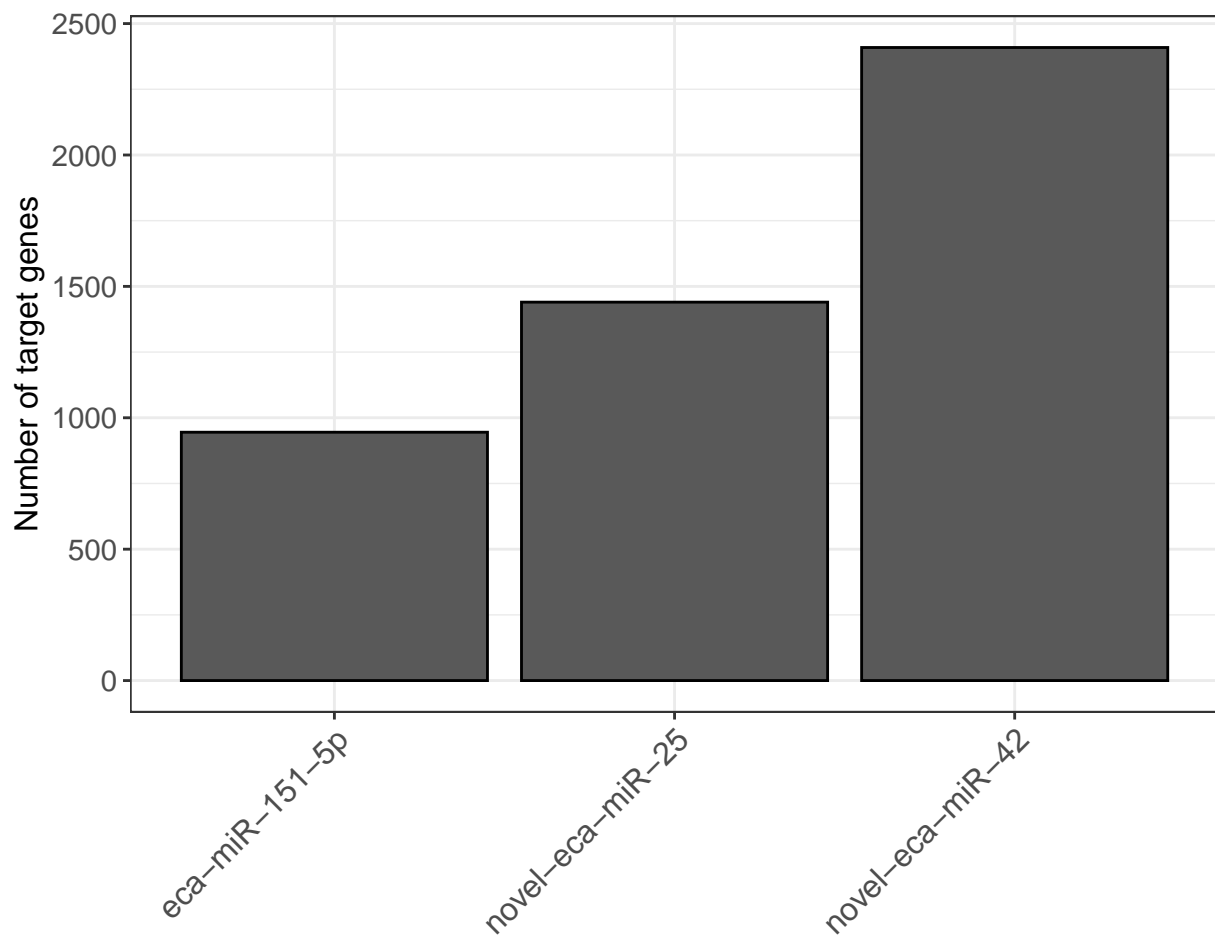

IIB vs. I

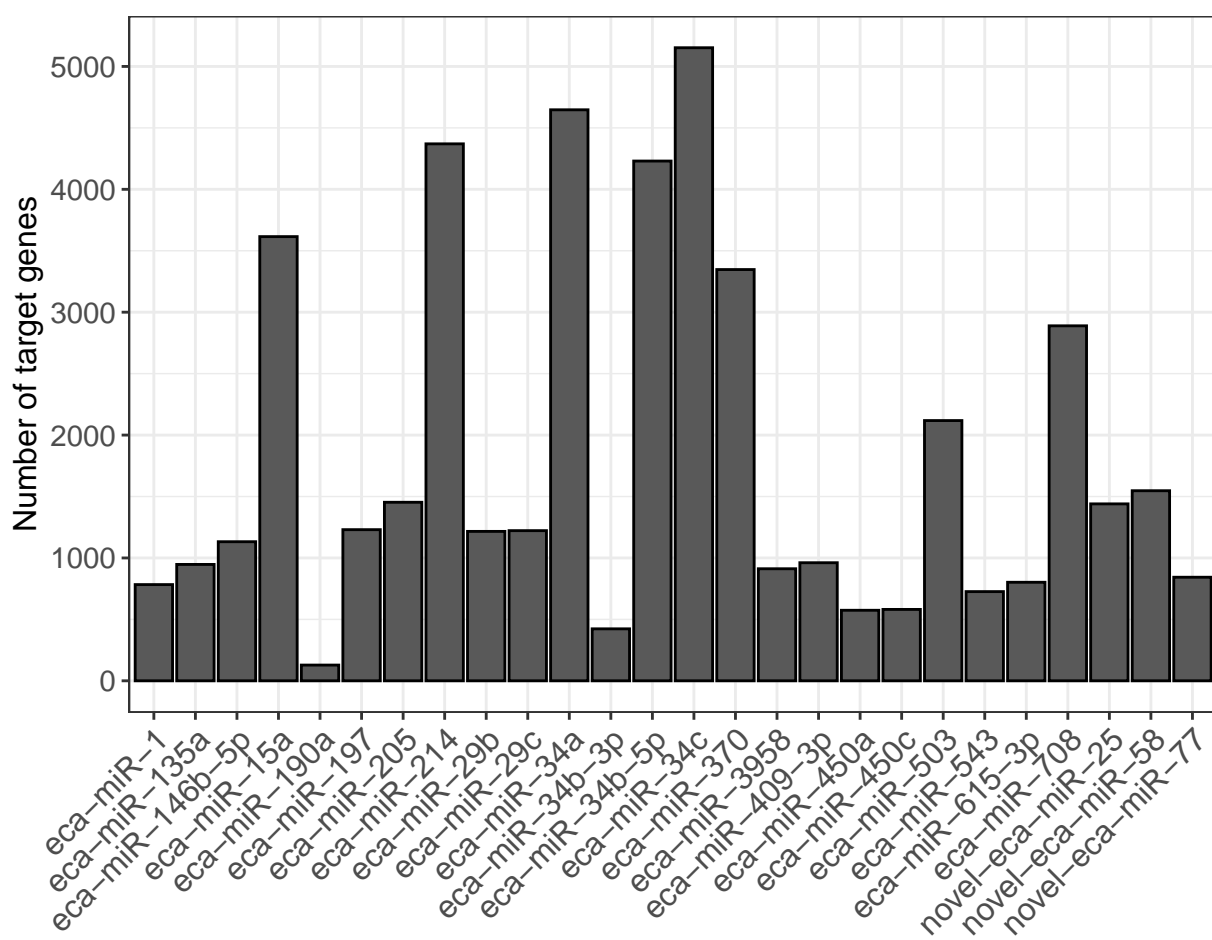

III vs. IIB

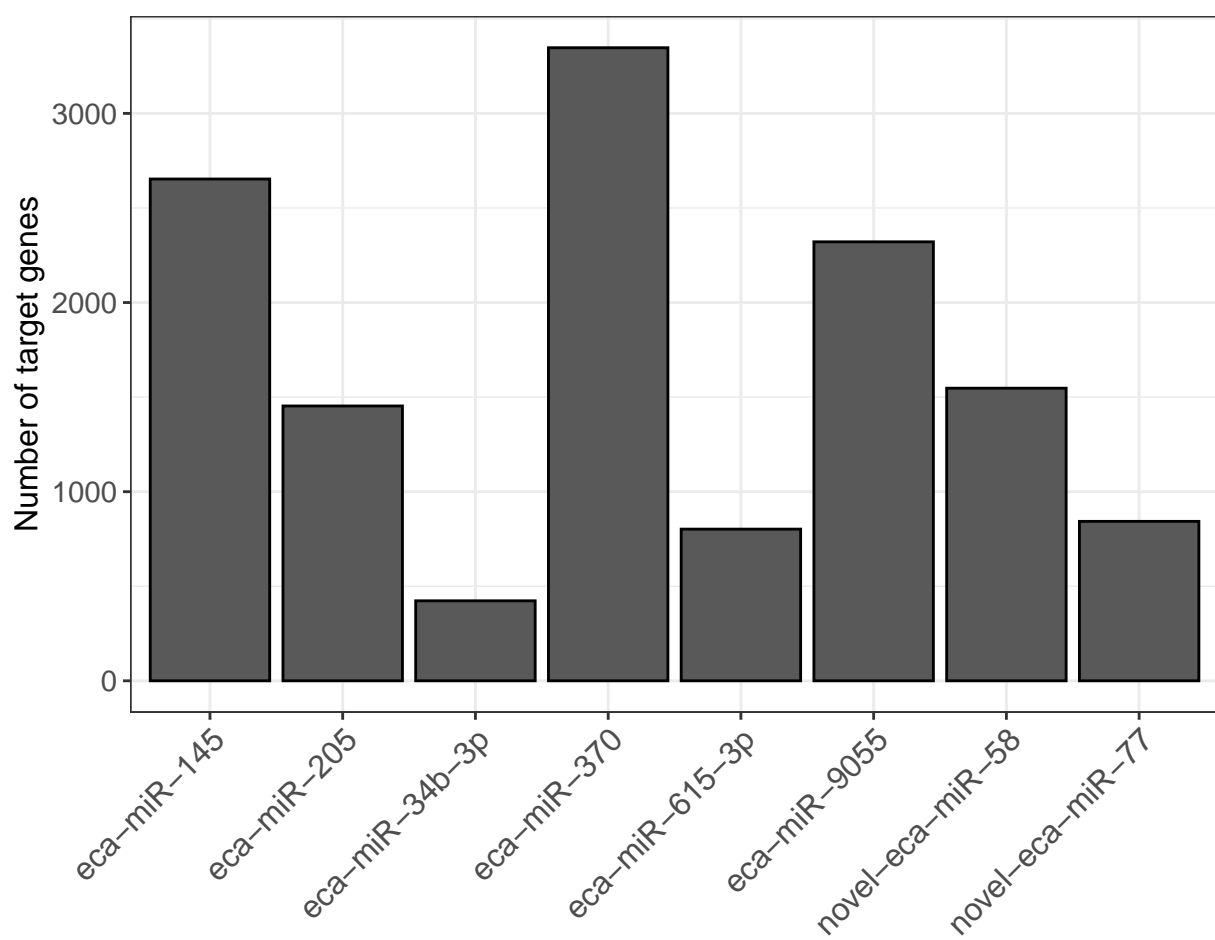

III vs. I

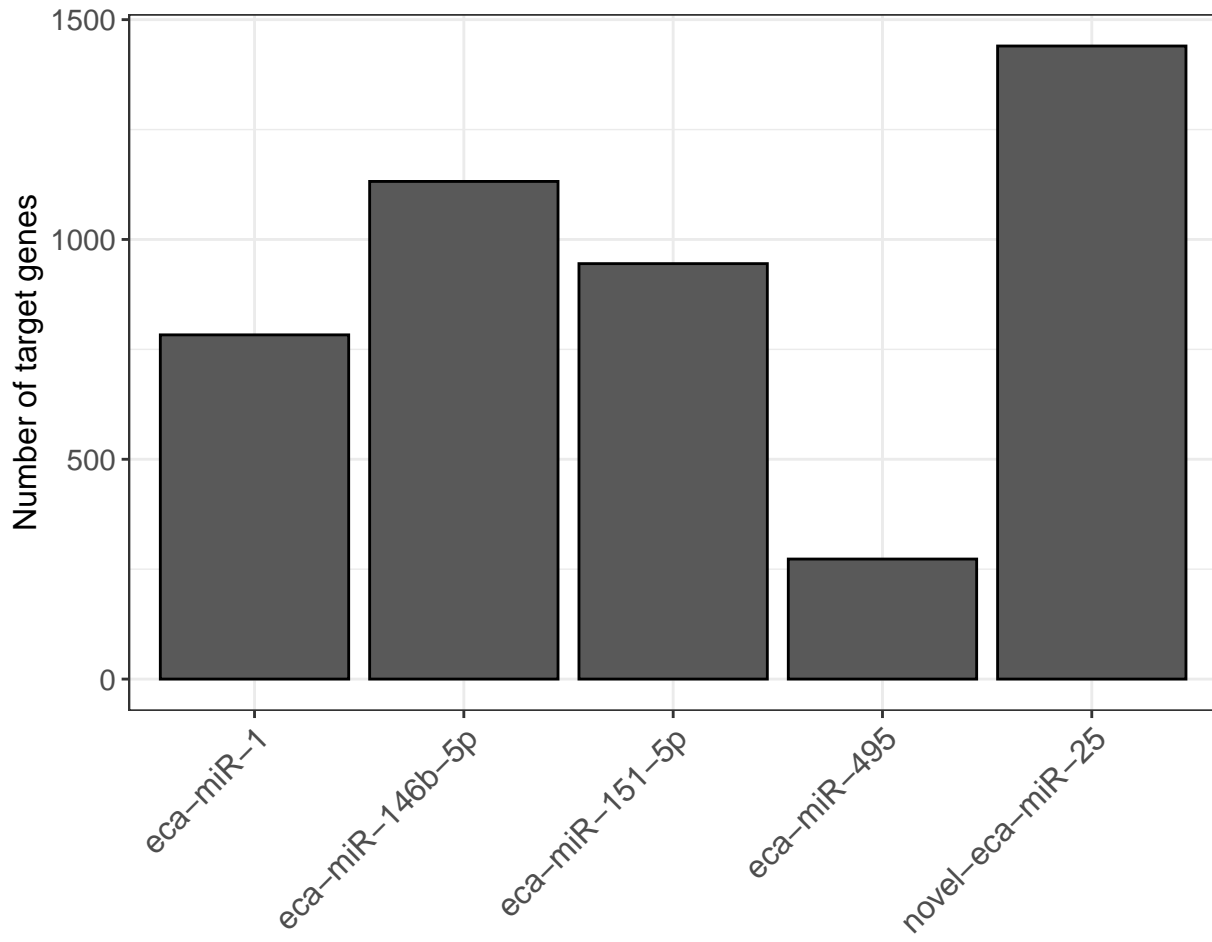

IIB vs. IIA

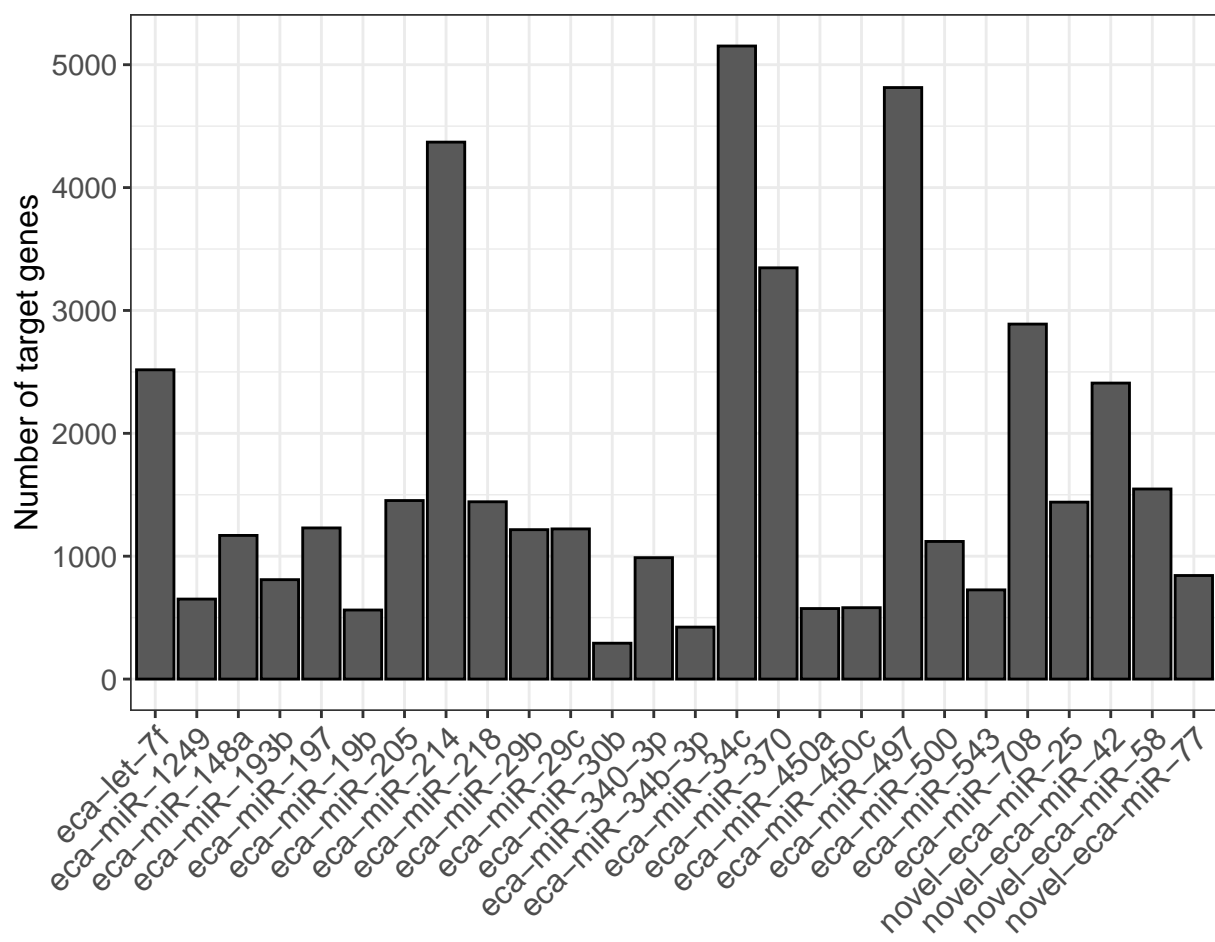

Supplement: Supplementary file 7 — Supplementary Information 6. [file 41598_2023_42149_MOESM7_ESM.pdf]

A)

I

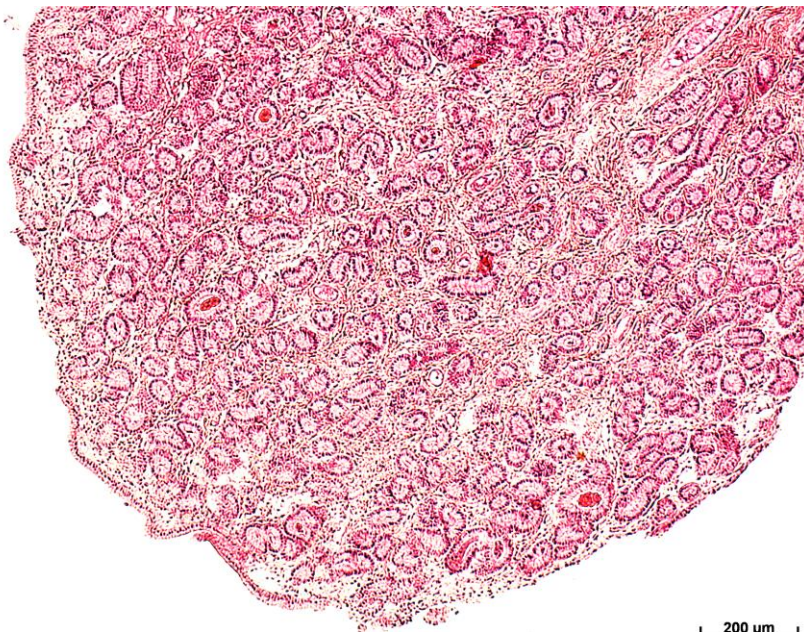

IIA

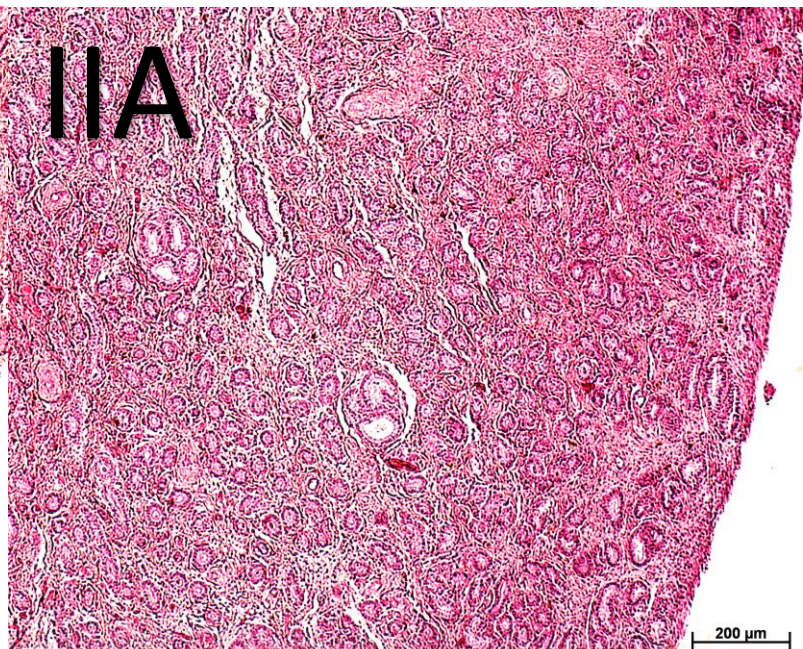

IIB

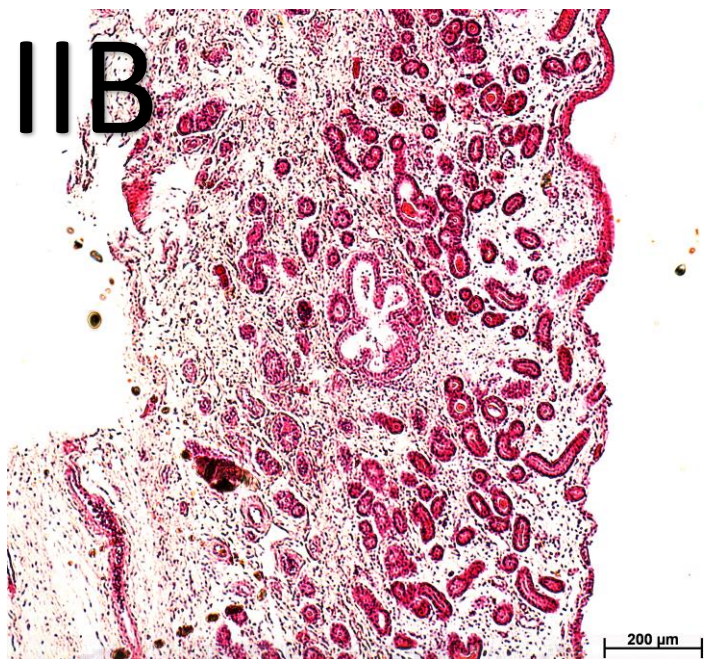

III

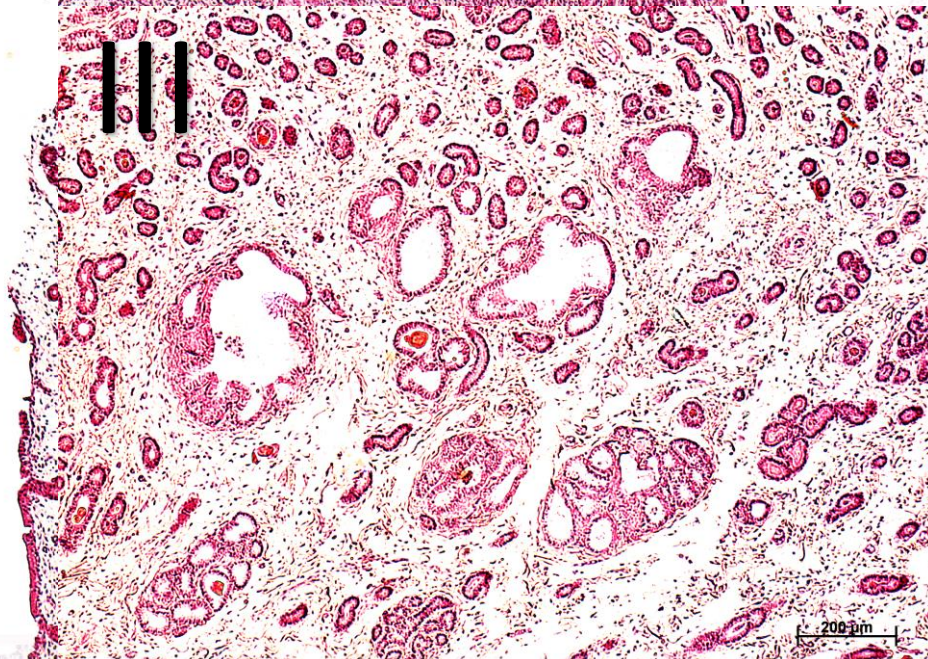

B)

I

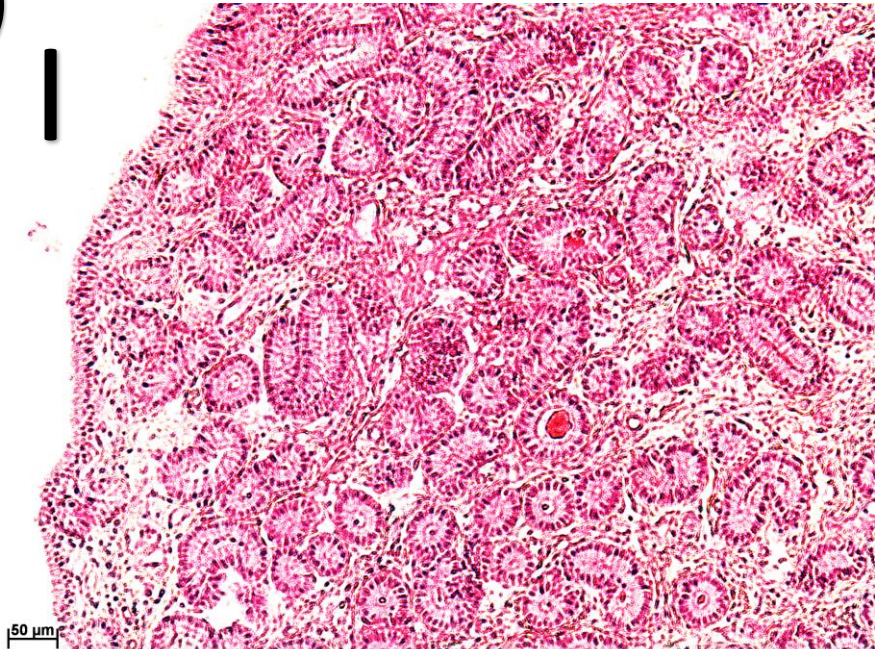

IIA

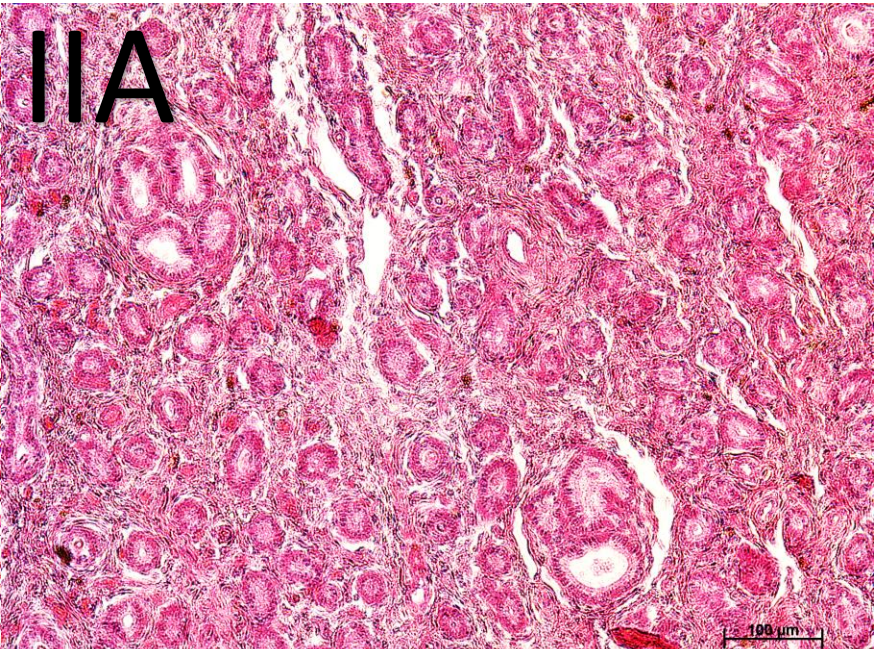

IIB

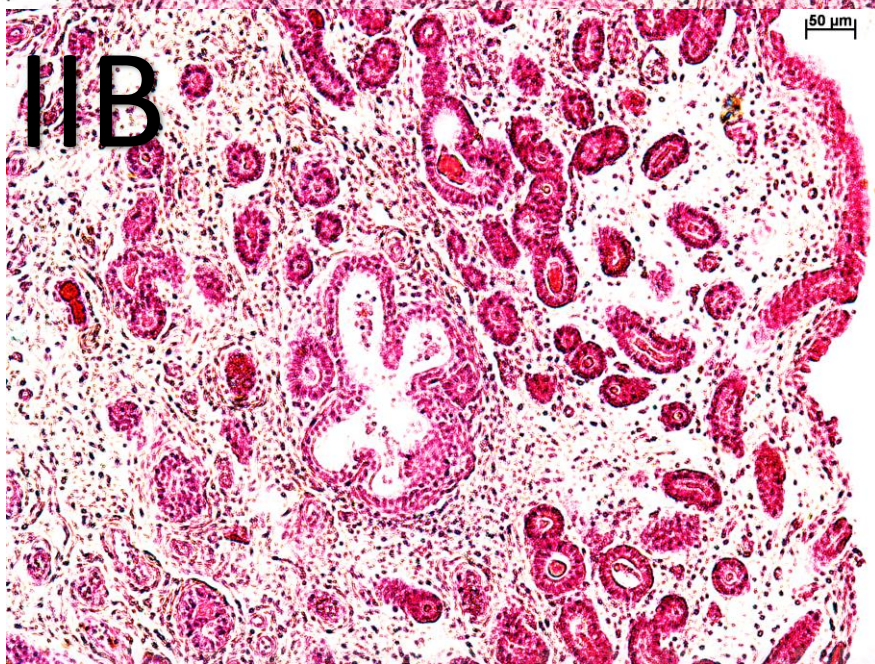

III

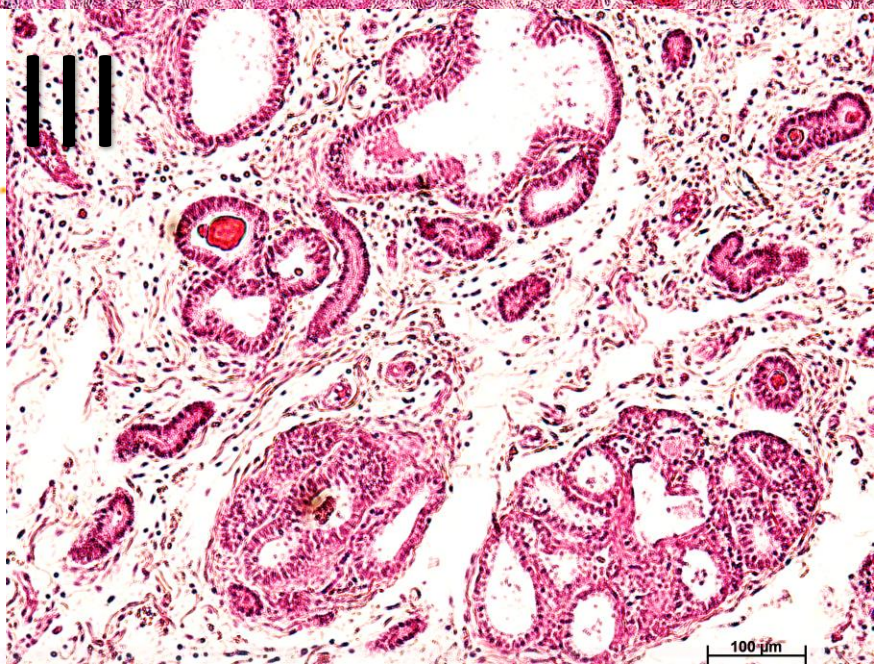

Supplement: Supplementary file 11 — Supplementary Information 10. [file 41598_2023_42149_MOESM11_ESM.pdf]
