# Supplementary material for: The potential role of miRNAs and regulation of their expression in the development of mare endometrial fibrosis
Source: Sci Rep. 2023 Sep 24;13:15938. doi: 10.1038/s41598-023-42149-3 (PMC10518347; doi:10.1038/s41598-023-42149-3)
Supplement: Supplementary file 3 — Supplementary Information 2. [file 41598_2023_42149_MOESM3_ESM.pdf]

Samples:	1-3	4-6	7-9	10-12	13-15	16-18	19-21	22-24	25-27	28-30	31-33	34-36	
Total number of	I			IIA			IIB			III			
reads before QC	3 251 144	26 304 055	9 201 737	21 895 391	3 577 676	9 564 410	5 135 055	8 333 899	20 436 622	59 899	7 908 930	2 650 974	
reads after QC	2 699 755	22 531 274	6 569 019	18 394 378	3 297 036	8 564 702	4 052 356	5 849 301	15 380 084	37 033	6 733 795	2 148 181	
ncRNA filtering	2 557 698	21 427 191	6 127 227	17 301 625	3 184 529	8 149 688	3 725 633	5 369 743	14 157 930	33 175	6 263 827	2 032 524	
mapped reads	1 997 080	17 028 846	4 557 590	13 457 491	2 621 427	6 413 481	2 789 190	3 939 342	10 199 843	24 555	4 828 396	1 605 439	
uniquely mapped reads	1 534 508	13 361 333	3 368 155	10 239 109	2 190 207	5 016 988	2 127 280	3 060 796	7 246 804	18 800	3 755 588	1 257 022	Total:
expressed known miRNAs	309	363	345	363	301	344	333	339	366	193	346	298	423
expressed novel miRNAs	25	49	36	63	24	36	33	40	48	3	48	19	90
