# Supplementary material for: The potential role of miRNAs and regulation of their expression in the development of mare endometrial fibrosis
Source: Sci Rep. 2023 Sep 24;13:15938. doi: 10.1038/s41598-023-42149-3 (PMC10518347; doi:10.1038/s41598-023-42149-3)
Supplement: Supplementary file 6 — Supplementary Information 5. [file 41598_2023_42149_MOESM6_ESM.pdf]

Comparison	No	DEmiR	Sequence	log2FC	P-adjusted
**IIA *vs.* I**	1	novel eca-miR-42	TTCTCAGGTTCGTCAGCCCATG	-27.18	5.27E-09
**IIB *vs.* I**	1	novel eca-miR-58	TACTCAGGGAGGCGTTGTTC	-24.05	1.56E-14
	2	noveleca-miR-77	TGGTGCAATTTTTGGAAAAA	-8.64	3.38E-04
	3	eca-miR-146b-5p	TGAGAACTGAATTCCATAGGCT	-1.61	1.03E-04
	4	eca-miR-370	GCCTGCTGGGGTGGAACCTGGT	-1.59	3.99E-06
	5	eca-miR-409-3p	GAATGTTGCTCGGTGAACCCCT	-1.45	5.98E-03
	6	eca-miR-543	AAACATTCGCGGTGCACTTCTT	-1.40	1.03E-04
	7	eca-miR-3958	CAGATATTGCACGGTTGATCTCTT	-1.33	1.03E-04
	8	eca-miR-1	TGGAATGTAAAGAAGTATGTAT	-1.28	1.97E-02
	9	eca-miR-503	TAGCAGCGGGAACAGTACTGCAG	-1.26	5.93E-03
	10	eca-miR-450c	TTTTGCGATGTGTTCCTAATAC	-1.10	1.98E-02
	11	eca-miR-450a	TTTTGCGATGTGTTCCTAATAT	-1.08	7.78E-03
	12	eca-miR-615-3p	TCCGAGCCTGGGTCTCCCTCTC	-1.03	7.85E-03
	13	eca-miR-197	TTCACCACCTTCTCCACCCAGC	1.06	3.11E-02
	14	eca-miR-15a	TAGCAGCACATAATGGTTTGTG	1.10	2.51E-02
	15	eca-miR-214	ACAGCAGGCACAGACAGGCAGT	1.17	2.24E-03
	16	eca-miR-135a	TATGGCTTTTTATTCCTATGTGA	1.32	8.53E-03
	17	eca-miR-34a	TGGCAGTGTCTTAGCTGGTTGT	1.33	3.38E-02
	18	eca-miR-29b	TAGCACCATTTGAAATCAGTGTT	1.39	1.45E-02
	19	eca-miR-34c	AGGCAGTGTAGTTAGCTGATTGC	1.41	1.98E-02
	20	eca-miR-34b-5p	AGGCAGTGTAATTAGCTGATTGT	1.42	3.79E-02
	21	eca-miR-708	AAGGAGCTTACAATCTAGCTGGG	1.55	5.93E-03
	22	eca-miR-34b-3p	AATCACTAACTCCACTGCCATC	1.60	2.32E-03
	23	eca-miR-29c	TAGCACCATTTGAAATCGGTTA	1.84	3.09E-02
	24	eca-miR-190a	TGATATGTTTGATATATTAGGT	2.53	2.47E-02
	25	eca-miR-205	TCCTTCATTCCACCGGAGTCTG	4.79	1.25E-04
	26	novel eca-miR-25	TACTCAGGGAGGCGTTGTTCA	9.18	2.32E-03
**III *vs.* I**	1	eca-miR-146b-5p	TGAGAACTGAATTCCATAGGCT	-1.53	3.71E-02
	2	eca-miR-495	AAACAAACATGGTGCACTTCTT	-1.29	3.24E-05
	3	eca-miR-1	TGGAATGTAAAGAAGTATGTAT	-1.10	-1.53E-05
	4	eca-miR-151-5p	TCGAGGAGCTCACAGTCTAGT	1.09	3.37E-02
	5	novel eca-miR-25	TACTCAGGGAGGCGTTGTTCA	8.97	3.71E-02
